# The effects of intermittent fasting on BMI, fasting blood glucose, and blood pressure in women with overweight or obesity: a systematic review and meta-analysis with dose–response relationships

**DOI:** 10.3389/fnut.2026.1818813

**Published:** 2026-05-14

**Authors:** Haoran He, Zhikai Qin, Kuiliang Liu, Zhitian Wang, Jiaoqin Wang

**Affiliations:** 1 Capital University of Physical Education and Sports, Beijing, China; 2 School of Physical Education and Sport Science, Provincial University Key Laboratory of Sport and Health Science, Fujian Normal University, Fuzhou, China; 3 Beijing No.54 Middle School, Dongcheng District, Beijing, China

**Keywords:** arterial pressure, Body Mass Index, dose–response correlation, Fasting blood glucose, periodic fasting, Systematic review, women with overweight or obesity

## Abstract

**Objective:**

This study aimed to assess the effects of intermittent fasting (IF) on prespecified clinically relevant outcomes, including BMI, fasting blood glucose (FBG), and blood pressure, in women with overweight or obesity, and to explore the dose–response relationship and moderating factors of the intervention.

**Methods:**

Following PRISMA guidelines, we systematically searched PubMed, Web of Science, PsycINFO, and the Cochrane Library (up to August 1, 2025) for eligible randomized controlled trials (RCTs). Twenty-two studies involving 1,287 women with overweight or obesity were included. Mean differences (MD) were pooled using a restricted maximum likelihood random-effects model (REML). Heterogeneity was evaluated via *I*^2^ and Q tests, while publication bias was assessed using sensitivity analyses, Egger's regression, and the trim-and-fill method. Subgroup analyses, meta-regression, and nonlinear dose–response models were used to identify moderators.

**Results:**

IF significantly reduced BMI in the final BMI model after exclusion of one influential study (MD = −0.41, 95% CI: −0.81 to−0.02, *P* = 0.0396) and FBG (MD = −2.18, 95% CI:−3.20 to−1.16, *P* < 0.0001) in women with overweight or obesity, with no significant effect on blood pressure. The initial BMI model showed substantial heterogeneity (*I*^2^ = 80.2%). Subgroup analyses suggested variability across intervention characteristics and participant strata, but these findings should be interpreted cautiously because several subgroup estimates were based on limited evidence. Dose–response analysis suggested a relatively favorable modeled frequency around twice weekly, with effects remaining relatively stable across 1,285–2,895 total intervention hours.

**Conclusion:**

IF may improve BMI and FBG in women with overweight or obesity, whereas no significant effects were observed for blood pressure outcomes. The dose–response findings suggest potentially favorable intervention patterns, but these should be interpreted cautiously because of heterogeneity across studies, variation in intervention and comparator conditions, possible co-interventions, and the limited certainty of the available evidence. Further well-designed trials are needed to confirm these findings and refine practical recommendations.

**Systematic Review Registration:**

https://www.crd.york.ac.uk/PROSPERO/view/CRD420251178671, identifier: CRD420251178671.

## Introduction

The global rise in obesity rates among women has emerged as a significant public health concern (1). Women with overweight or obesity frequently exhibit higher body mass index (BMI), abnormal fasting blood glucose (FBG) levels, and elevated blood pressure. These conditions are interrelated and significantly heighten the likelihood of developing type 2 diabetes and heart-related illnesses (2–4). Research indicates that around 60% of women with overweight or obesity experience issues with FBG, while 45% suffer from high blood pressure.

In recent times, the primary strategies for managing obesity have included ongoing energy-restricted diets, consistent physical activity, and behavioral therapy (5–7). While these methods have formed the foundation of weight control, each encounters unique obstacles: ongoing energy restriction can successfully lower body weight but often suffers from low long-term compliance, leading to a tendency toward weight regain (5). Regular physical activity is vital for enhancing metabolic health, yet it can be hindered by limitations such as time constraints, physical ability, or joint stress (6). Additionally, the success of behavioral therapy may have plateaued due to variations among individuals and psychosocial influences (7).

Intermittent fasting (IF) is characterized by alternating periods of fasting and eating and has attracted increasing attention as a dietary strategy for metabolic regulation, with prior reviews summarizing its potential effects on body weight, glycemic control, and cardiometabolic health across different fasting regimens (4, 8, 9). Key mechanisms of IF include extended fasting periods that enhance fat burning, boost insulin sensitivity, and align circadian rhythms for better blood pressure control (10–13). Women, in particular, have embraced IF more than continuous energy restriction (CER) due to its adaptable nature and improved long-term compliance (3, 8, 14). For example, time-restricted eating (TRE), which typically limits the daily eating window to approximately 6–12 h, with common schedules including 18:6, 16:8, 14:10, and 12:12, allows for reduced caloric intake without the need for strict calorie counting, making it more appealing than conventional calorie restriction (15). Both experimental and clinical studies indicate that IF offers various metabolic benefits, with adherence and weight-loss outcomes similar to those of CER, along with distinct advantages (16, 17). Research has demonstrated that the effects of IF on metabolic markers depend on the specific pattern used. TRE and alternate-day fasting (ADF) have been effective in lowering BMI, with a 12-week program resulting in weight and BMI reductions among individuals with obesity (1, 14, 18). In terms of glucose regulation, both TRE and ADF have been shown to decrease fasting insulin and enhance insulin sensitivity, with some findings suggesting these benefits occur regardless of weight fluctuations (3, 12, 19). Additionally, IF has been linked to reductions in both systolic and diastolic blood pressure, especially in those with hypertension, and this improvement may be closely associated with weight loss (10, 11, 20).

The current understanding of IF and its impact on health metrics in women is still quite limited. Most research on IF has primarily concentrated on male subjects or mixed-gender groups. Given differences in hormonal profiles, body fat distribution, and fasting-related metabolic responses between sexes, women's responses to IF may differ from those of men (21, 22). Previous systematic reviews of Ramadan intermittent fasting (RIF) have also suggested that sex may influence fasting-related anthropometric, biochemical, and disease-related outcomes (23, 24). Accordingly, the present review focused specifically on women to reduce sex-related clinical heterogeneity and improve the interpretability of pooled estimates. For instance, studies indicate that postmenopausal women with overweight or obesity experience less weight and body fat loss from TRE compared to their male counterparts (22). Additionally, the influence of different IF approaches on BMI, FBG, and blood pressure in women remains inadequately explored, particularly TRE regimens such as 16:8 schedules, the subtype eTRE, and ADF. Some research suggests that eTRE may be more effective than other methods in enhancing insulin sensitivity and managing blood pressure (12, 21). Furthermore, many existing studies involve small participant groups and typically have follow-up durations of less than 6 months, leaving the long-term implications and safety of these fasting approaches unverified (1, 25, 26).

Numerous systematic reviews and meta-analyses have validated the metabolic advantages of IF from various angles. An umbrella review revealed that IF provides robust evidence for enhancing waist circumference, body fat percentage, fasting insulin levels, and low-density lipoprotein cholesterol in adults with overweight or obesity; however, its impact on lowering systolic blood pressure (SBP) was found to be less significant than that of CER (25). This observation aligns with a meta-analysis focused on TRE, which indicated that the extent of blood pressure reduction associated with TRE may depend on factors such as the length of the intervention (11). In addition, previous systematic reviews of RIF have reported small reductions in body weight and favorable changes in selected glucometabolic markers in generally healthy populations, further suggesting that the metabolic effects of fasting may vary according to fasting model, population characteristics, and study design (27–29). Nevertheless, despite the increasing evidence, the majority of studies have predominantly included male subjects or did not differentiate participants by gender, suggesting that the understanding of IF's effects on women remains limited. Additionally, the optimal parameters of major IF approaches in women, particularly TRE-based regimens and ADF, remain insufficiently defined (30). This underscores the necessity for more research into the metabolic benefits of IF in women with overweight or obesity, particularly in understanding the dose–response relationships within specific demographic groups (31). Therefore, we performed a systematic review and a dose-response meta-analysis to accurately assess the overall impact of IF on BMI, FBG levels, and blood pressure in women with overweight or obesity, with a focus on factors such as frequency of intervention, duration of fasting, and age. The findings aim to support informed decision-making for interventions in this population.

## Materials and methods

### Study design

This paper offers a comprehensive analysis of randomized controlled trials (RCTs) conducted according to the guidelines of the Preferred Reporting Items for Systematic Reviews and Meta-Analyses (PRISMA) (32). Before evaluating the search outcomes, the review's protocol was documented in the International Prospective Register of Systematic Reviews (PROSPERO) under registration number CRD420251178671, and the study's findings complied with the PRISMA statement.

### Study inclusion criteria

The criteria for participation in this research were established as follows: The study utilized an RCT framework to evaluate the impact of IF on women with overweight or obesity; The intervention group engaged in specific intermittent fasting models, including TRE/TRF (time-restricted eating/time-restricted feeding), e.g., 12:12, 14:10, 16:8, and 18:6 schedules, eTRE, ADF/ADMF, and intermittent energy restriction (IER) approaches, including 2-day-per-week regimens and 5:2-type schedules. One 12-h daily fasting protocol was classified under TRF rather than treated as a separate extended overnight fasting model, while the comparator group followed calorie-restriction-based diets (e.g., continuous calorie restriction, daily calorie restriction, or hypocaloric diet), standard diets/usual care, or placebo/control conditions, depending on the original trial design; Participants were female individuals aged 18 years or older, with a BMI of 25 kg/m^2^ or higher, consistent with the prespecified overweight-or-obesity eligibility scope, with no further demographic limitations. This sex-specific eligibility criterion was prespecified to maintain a clinically more homogeneous population, because hormonal status, reproductive stage, and obesity-related metabolic characteristics differed substantially across the female populations represented in the included literature; All relevant literature had to be published in English and available in full text. Exclusion criteria encompassed: male participants, those younger than 18, individuals with severe health conditions (such as type 1 diabetes, severe heart disease, cancer, or significant liver and kidney issues) that could influence results, non-English publications, and studies lacking full-text access; additionally, descriptive reviews, preclinical investigations, duplicate articles, editorials or opinion pieces, gray literature, and conference proceedings were excluded. Systematic reviews and study protocols that did not meet the inclusion criteria were included for reference and cited as needed. Although RIF is conceptually a form of IF and prior systematic reviews have reported modest body-weight reduction and favorable changes in selected glucometabolic markers during Ramadan fasting (28, 29), it was not prespecified as an eligible intervention model for quantitative synthesis because the present review was restricted to randomized controlled dietary interventions with planned intervention delivery and comparator groups, whereas much of the RIF literature falls outside this prespecified study-design scope.

### Search strategy

This research conducted a thorough search of the PubMed, Web of Science, PsycINFO, and Cochrane Library databases up to August 1, 2025, to identify eligible RCTs. The search employed the PICOS framework, targeting women with overweight or obesity as the population (*P*), specific intermittent fasting models as the intervention (*I*), including time-restricted eating/time-restricted feeding (TRE/TRF; e.g., 12:12, 14:10, 16:8, and 18:6 schedules), early time-restricted eating (eTRE), alternate-day fasting or alternate-day modified fasting (ADF/ADMF), and intermittent energy restriction approaches, including 2-day-per-week regimens and 5:2-type schedules. Extended overnight fasting-related terms were additionally included in the updated search strategy for completeness, although no eligible study was ultimately classified as a separate extended overnight fasting model in the present review. Standard dietary practices or the absence of specific dietary changes were treated as the control (C), and the primary outcomes were alterations in BMI, FBG, and blood pressure (both systolic and diastolic) (O), with RCTs as the study design (S). A combination of keywords and subject terms was utilized, including intermittent fasting-related terms (e.g., intermittent fasting, time-restricted eating, time-restricted feeding, early time-restricted feeding, alternate-day fasting, alternate-day modified fasting, intermittent energy restriction, 5:2, extended overnight fasting, overnight fasting, and 12-h fasting), population-related terms for women with overweight or obesity, outcome-related terms, and randomized trial terms. The detailed search methodology for each database is available in the Supplementary document. The outcome set was prespecified to focus on three clinically interpretable domains that are commonly used in trials of IF in women with overweight or obesity: BMI as an anthropometric indicator of overall weight status, FBG as an indicator of basal glycemic regulation, and systolic / diastolic blood pressure (DBP) as indicators of hemodynamic status. These outcomes were selected because they are clinically relevant, routinely reported, and generally comparable across studies. We acknowledge that other related outcomes, such as fasting insulin, HOMA-IR, waist circumference, body fat percentage, and other anthropometric or metabolic markers, may provide additional information. However, the present review was designed as a focused synthesis of prespecified outcomes rather than a comprehensive evaluation of all obesity-related metabolic indicators.

### Study selection process

All retrieved records were imported into Zotero 7.0 for automated duplicate detection and removal. In addition, an automation-assisted pre-screening step was used to identify clearly ineligible records before formal title/abstract screening. Records removed for other documented reasons before title/abstract screening were summarized separately. Subsequently, two reviewers independently screened titles and abstracts, and potentially eligible reports were retrieved for full-text assessment.

### Data synthesis

The statistical analyses conducted in this research used R 4.3.3, focusing on packages such as meta, metafor, diamet, and ggplot2. For the analysis of continuous outcomes, either the mean difference (MD) or the standardized mean difference (SMD) was selected as the effect size, contingent on the uniformity of the measurement scales across the studies. The effect size was reported as the mean difference (MD), the average difference between the intervention and control groups in their original measurement units. Positive and negative values indicated increases or decreases in the intervention's effect, with clinical significance interpreted based on the specific outcome variable's actual units (33). The overall effect size was computed using the inverse-variance weighting approach, primarily via a restricted maximum likelihood (REML) random-effects model. In instances of low heterogeneity (*I*^2^ < 50%), results from a fixed-effect model were also included. The assessment of heterogeneity involved: 1) the Q test (with *P* < 0.10 deemed significant); 2) the *I*^2^ index (values over 50% indicating substantial heterogeneity) (34). Publication bias was evaluated using funnel plots and Egger's regression test, and any asymmetry was adjusted using the trim-and-fill technique (35). In model diagnostics, influential outliers were identified using standardized residuals (|Z| > 2.5) and Cook's distance (greater than 3 times the mean) (36). To further confirm the reliability of the findings, several sensitivity analyses were conducted: (1) sequential removal of items (using the Metainf function); (2) meta-regression analysis via the REML method to investigate the relationship between potential moderating factors (like intervention duration and population type) and effect sizes, with regression trends illustrated through bubble plots (37); (3) subgroup stratification tests to identify sources of heterogeneity. To ensure a consistent analytical framework, the mean difference (MD) was employed as the effect size, with its calculation formula detailed $MD=M_{Intervention}-M_{control},SD_{pooled}=\sqrt{\frac{\left(n_{1}-1\right)SD_{1}^{2}+\left(n_{2}-1\right)SD_{2}^{2}}{n_{1}+n_{2}-2}}\;$ (38).

### Risk of bias (Quality) assessment

The 2019 updated ROB2 tool from the Cochrane Collaboration was employed in this research to evaluate bias risk across all RCTs included in the analysis. The evaluation criteria encompassed: 1) the randomization process, 2) deviations from the intended intervention, 3) absence of outcome data, 4) measurement of outcomes, and 5) bias in selective reporting (39). Two researchers independently conducted the review, categorizing each trial as “low risk,” “some concerns,” or “high risk”. In instances of disagreement, discussions were held internally, and if a consensus was not achieved, a third reviewer was brought in to mediate. The overall confidence in the evidence regarding the most effective intervention was determined using the GRADE methodology, with the evaluation of evidence certainty following the GRADE framework (40).

## Results

### Study selection

**Figure 1:**
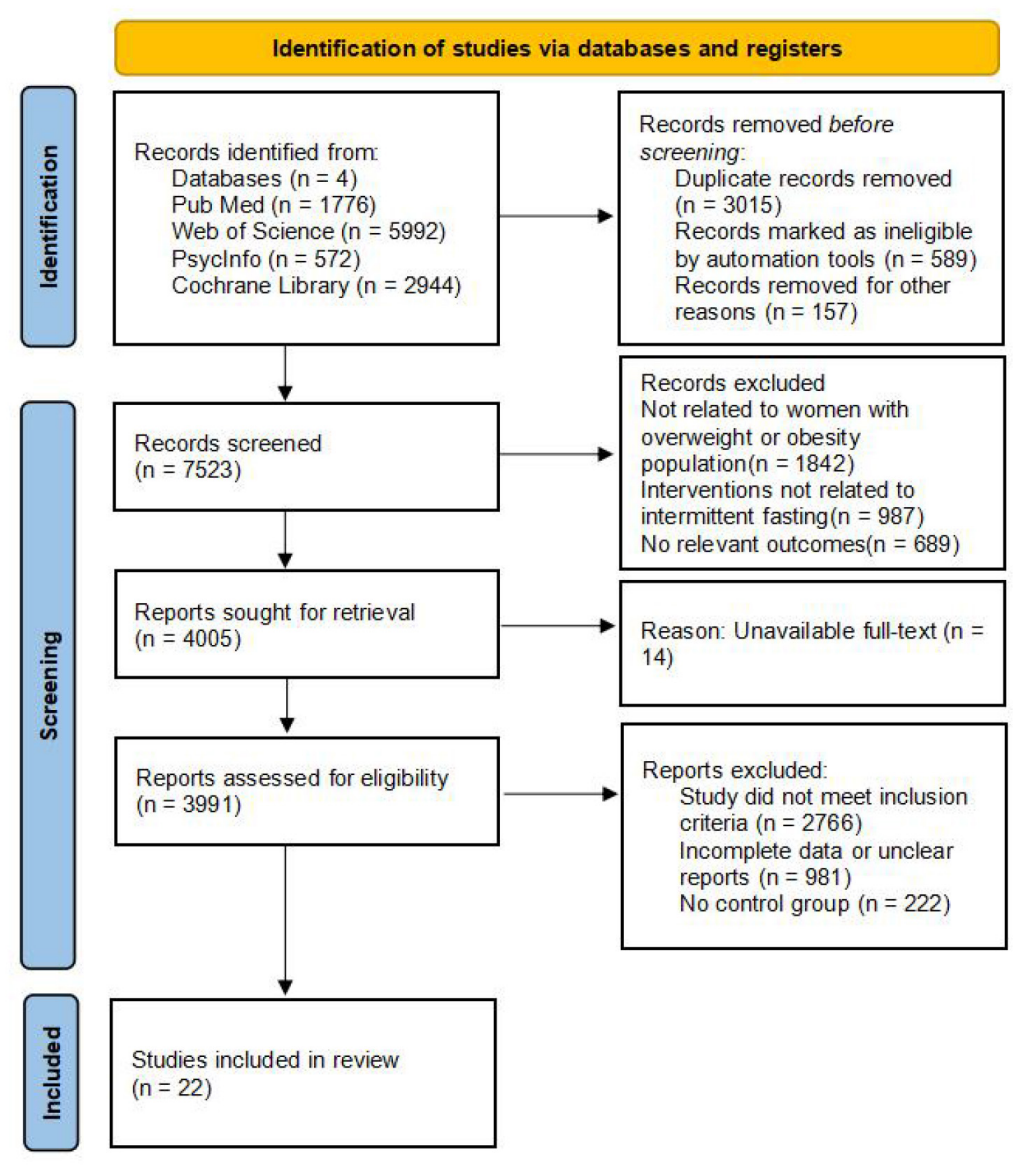
Flow diagram of the selection process.

A total of 11,284 records were identified from PubMed (*n* = 1,776), Web of Science (*n* = 5,992), PsycINFO (*n* = 572), and the Cochrane Library (*n* = 2,944). After removal of 3,015 duplicate records, 589 records marked as ineligible by automation tools, and 157 records removed for other documented reasons before title/abstract screening, 7,523 records were screened. Of these, 3,518 records were excluded because they were not related to women with overweight or obesity (*n* = 1,842), did not evaluate intermittent fasting interventions (*n* = 987), or did not report relevant outcomes (*n* = 689). The full texts of 4,005 reports were sought for retrieval, of which 14 could not be obtained. Therefore, 3,991 reports were assessed for eligibility. During full-text review, 3,969 reports were excluded because they did not meet the inclusion criteria (*n* = 2,766), contained incomplete data or unclear reports (*n* = 981), or lacked an eligible control group (*n* = 222). The specific reasons for these exclusions are illustrated in Figure 1. Detailed reasons for records removed for other documented reasons before title/abstract screening and for full-text exclusions are provided in Supplementary Tables S1, S2. Ultimately, 22 studies were included in the systematic review (41–62).

### Study characteristics

Table 1 presents an overview of the key features of 22 RCTs, which included 1,287 participants in total. These investigations covered several IF models, including TRE/TRF/eTRE, ADMF, and multiple 2-day-per-week intermittent restriction protocols that were operationally consistent with 5:2-type approaches. Two included 12-h daily fasting protocols overlapped conceptually with extended overnight fasting, but they were classified under TRF in the present review. These investigations focused on the impact of IF on various health metrics, including weight, FBG levels, and systolic and diastolic blood pressure, among women with overweight or obesity. The studies were evaluated according to their research goals, design elements, outcome indicators, and significant results.

**Table 1:** Characteristics of the studies in the systematic review and meta-analysis.

Reference	Country	Design	Sample (T/C)	Mean age (years)	Subject type	Intervention (T)	Comparator (C)	Protocol	Outcomes assessed
Batitucci (44)	Brazil	parallel RCT	15/10/11	32.2	women with obesity	5:2 IF plus HIIT/5:2 IF alone	HIIT alone	18 h/session, 2 × /week, 8 weeks	BMI
Beaulieu (50)	United Kingdom	parallel RCT	12/18	34.5	women with obesity	IER	CER	16 h/session, 2 × /week, 12 weeks	BMI
de Oliveira Maranhão Pureza IR (48)	Brazil	parallel RCT	31/27	31.4	women with obesity	HD+TRF	HD	12 h/session, 7 × /week, 52 weeks (conceptually overlapping with extended overnight fasting)	BMI, Diastolic BP, Systolic BP
Domaszewski (54)	Poland	parallel RCT	25/20	65	women with overweight or obesity	TRE	UDC	16 h/session, 7 × /week, 6 weeks	BMI
Domaszewski (55)	Poland	parallel RCT	29/28	69.2	women with overweight or obesity	TRE	EC	16 h/session, 7 × /week, 6 weeks	BMI
Fagundes (43)	Brazil	parallel RCT	24/12	34.5	women with overweight or obesity	TRE	CR–noTR	16 h/session, 7 × /week, 8 weeks	BMI, FBG
Gray (51)	Australia	parallel RCT	29/30	39.6	women with overweight	2–day/week IER	CER	16 h/session, 2 × /week, 52 weeks	BMI
Haganes (49)	Norway	parallel RCT	33/33	36.2	women with overweight or obesity	TRE	NIC	10 h/session, 7 × /week, 7 weeks	BMI, FBG, Diastolic BP, Systolic BP
Harvie (52)	United Kingdom	parallel RCT	53/54	40.0	women with overweight or obesity	2–day/week IER	CER	24 h/session, 2 × /week, 26 weeks	BMI, Diastolic BP, Systolic BP
Harvie (46)	United Kingdom	parallel RCT	37/38/40	47.3	women with overweight	2–day/week IECR	IECR+PF/CER	24 h/session, 2 × /week, 17 weeks	BMI, FBG, Diastolic BP, Systolic BP
Hooshiar (57)	Iran	parallel RCT	28/28	35.58	women with overweight or obesity	ADMF	CR	22 h/session, 3 × /week, 8 weeks	BMI
Hooshiar (58)	Iran	parallel RCT	25/24	31.94	women with overweight or obesity	ADMF	DCR	22 h/session, 3 × /week, 8 weeks	BMI
Hutchison (41)	Australia	parallel RCT	51/12	50	women with overweight	3–day/week 24–h IF	CER	24 h/session, 3 × /week, 8 weeks	BMI, FBG
Irani (45)	Iran	parallel RCT	29/27	42.32	women with overweight or obesity	TRF	CER	14 h/session, 7 × /week, 8 weeks	BMI
Keawtep (56)	Thailand	parallel RCT	21/20	52.8	women with obesity	2–day/week IF	NIC	16 h/session, 2 × /week, 12 weeks	BMI
Lin (62)	China	parallel RCT	30/33	52.3	women with overweight or obesity	TRE	UDC	16 h/session, 7 × /week, 8 weeks	BMI, FBG, Diastolic BP, Systolic BP
Pureza (47)	Brazil	parallel RCT	31/21	31.4	women with obesity	HD+TRF	HD	12 h/session, 7 × /week, 3 weeks (conceptually overlapping with extended overnight fasting)	BMI, FBG, Diastolic BP, Systolic BP
Ranjbar (53)	Iran	parallel RCT	22/22	57.2	women with overweight or obesity	16:8 daily IF	UDC	16 h/session, 7 × /week, 8 weeks	BMI
Salma (42)	Jordan	parallel RCT	57/29	35.1	women with obesity	3–day/week 18–h IF + DR	DR	18 h/session, 3 × /week, 6 weeks	BMI
Schroder (59)	Brazil	parallel RCT	20/12	38.9	women with obesity	TRE	UDC	16 h/session, 7 × /week, 17 weeks	BMI, FBG, Diastolic BP, Systolic BP
Talebi (60)	Iran	parallel RCT	30/30/30	30.3	women with obesity	eTRE+Probiotic	eTRE+Placebo/ DCR+Placebo	14 h/session, 7 × /week, 8 weeks	BMI, FBG
Teong (61)	Australia	parallel RCT	22/24	50	women with overweight or obesity	IER	CER	24 h/session, 3 × /week, 8 weeks	BMI

RCT, randomized controlled trial; T, intervention group; C, comparator group; IF, intermittent fasting; EX, exercise; HIIT, high-intensity interval training; TRE, time-restricted eating; TRF, time-restricted feeding; eTRE, early time-restricted eating; IER, intermittent energy restriction; IECR, intermittent energy and carbohydrate restriction; PF, protein and fat; ADMF, alternate-day modified fasting; CER, continuous calorie restriction; CR, calorie restriction; DCR, daily calorie restriction; HD, hypocaloric diet; UDC, usual-diet control; EC, educational control; CR-noTR, calorie restriction without time restriction; NIC, non-intervention control; BMI, body mass index; FBG, fasting blood glucose; SBP, systolic blood pressure; DBP, diastolic blood pressure. UDC was used as a standardized label for comparator conditions originally described as habitual diet or usual diet. Specific IF subtypes (e.g., TRE, TRF, eTRE, IER, IECR, and ADMF) were reported in Table 1 whenever explicitly described in the original trials. Generic “IF” was avoided in Table 1 whenever the original protocol allowed a more specific operational description (e.g., 16:8 daily IF, 24-h IF 3 × /week, 2-day/week IF, 5:2 diet, 2-day/week IER, or 2-day/week IECR). The included 12-h daily fasting protocols were classified under TRF rather than treated as a separate extended overnight fasting model.

### Risk of bias of included studies

**Figure 2:**
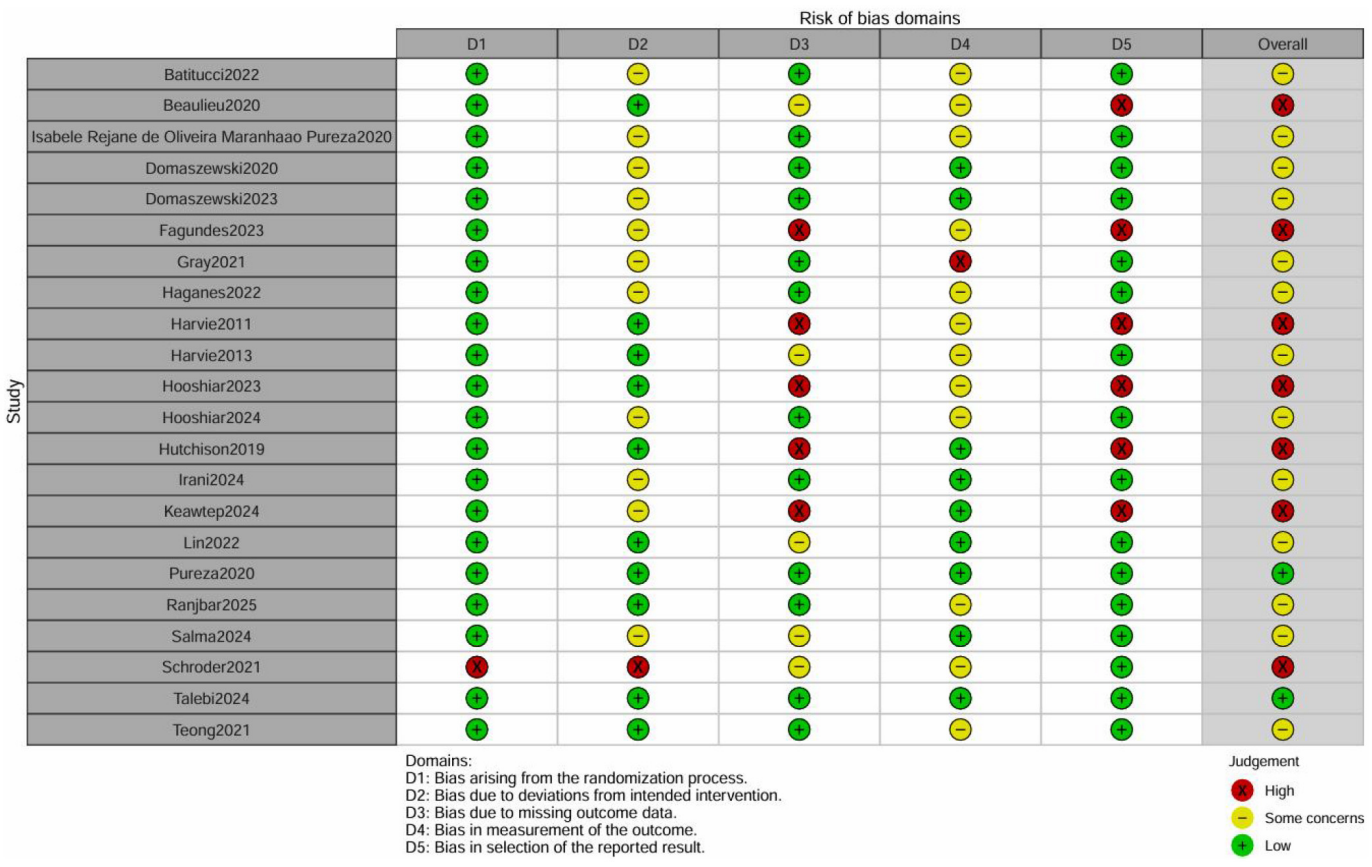
Risk of bias summary.

**Figure 3:**
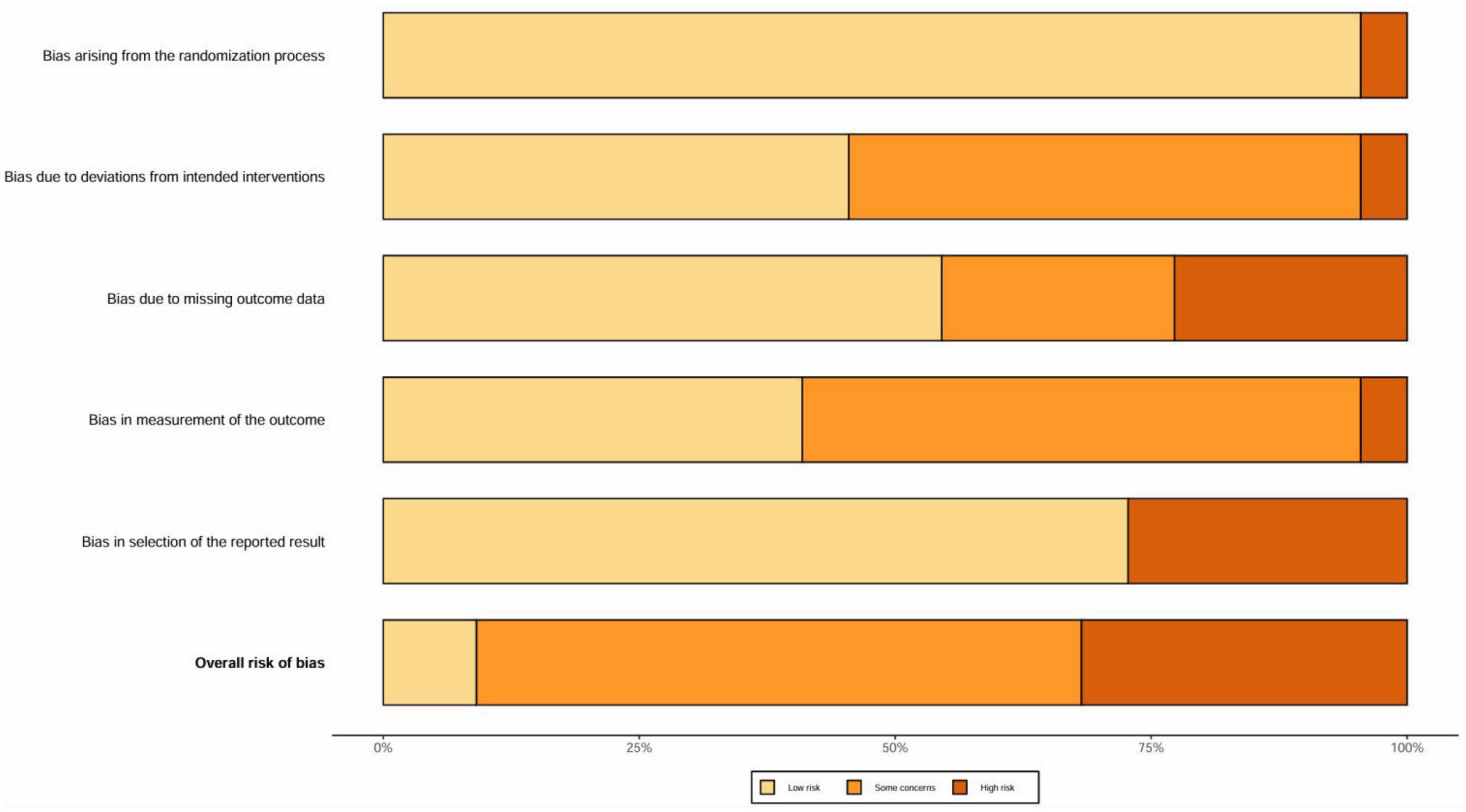
Risk of bias bar plot.

Every randomized controlled trial (RCT) included in this analysis confirmed the use of random allocation, providing comprehensive details on the methods for generating random sequences (such as computer-generated randomization and random number tables) and the mechanisms for concealing allocation (such as sealed envelopes). Consequently, the randomization process was deemed to have a low risk. Regarding data completeness, eight studies documented their loss-to-follow-up rates and employed intention-to-treat (ITT) analysis. The potential for loss-to-follow-up bias was effectively managed, with 14 studies presenting complete datasets (41–44, 47, 49–53, 55, 58, 62). Additionally, 12 studies thoroughly reported their predefined outcomes (41–43, 46, 47, 50–53, 55, 58, 59, 61, 62). Figures 2, 3 illustrate a Risk of Bias Summary Table and a Risk of Bias Bar Plot, respectively.

### Meta-analysis

The research conducted a thorough evaluation of how IF impacts BMI, FBG levels, and blood pressure among women with overweight or obesity, as summarized in Figure 7.

**Figure 4:**
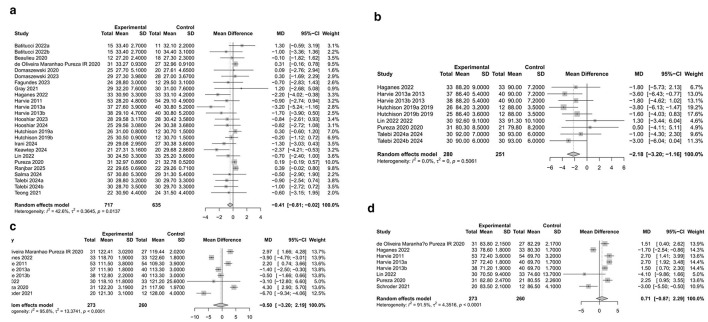
**(A)** Forest plot of the effect of IF on BMI in women with overweight or obesity. **(B)** Forest plot of the effect of IF on FBG in women with overweight or obesity. **(C)** Forest plot of the effect of IF on SBP in women with overweight or obesity. **(D)** Forest plot of the effect of IF on DBP in women with overweight or obesity.

**Figure 5:**
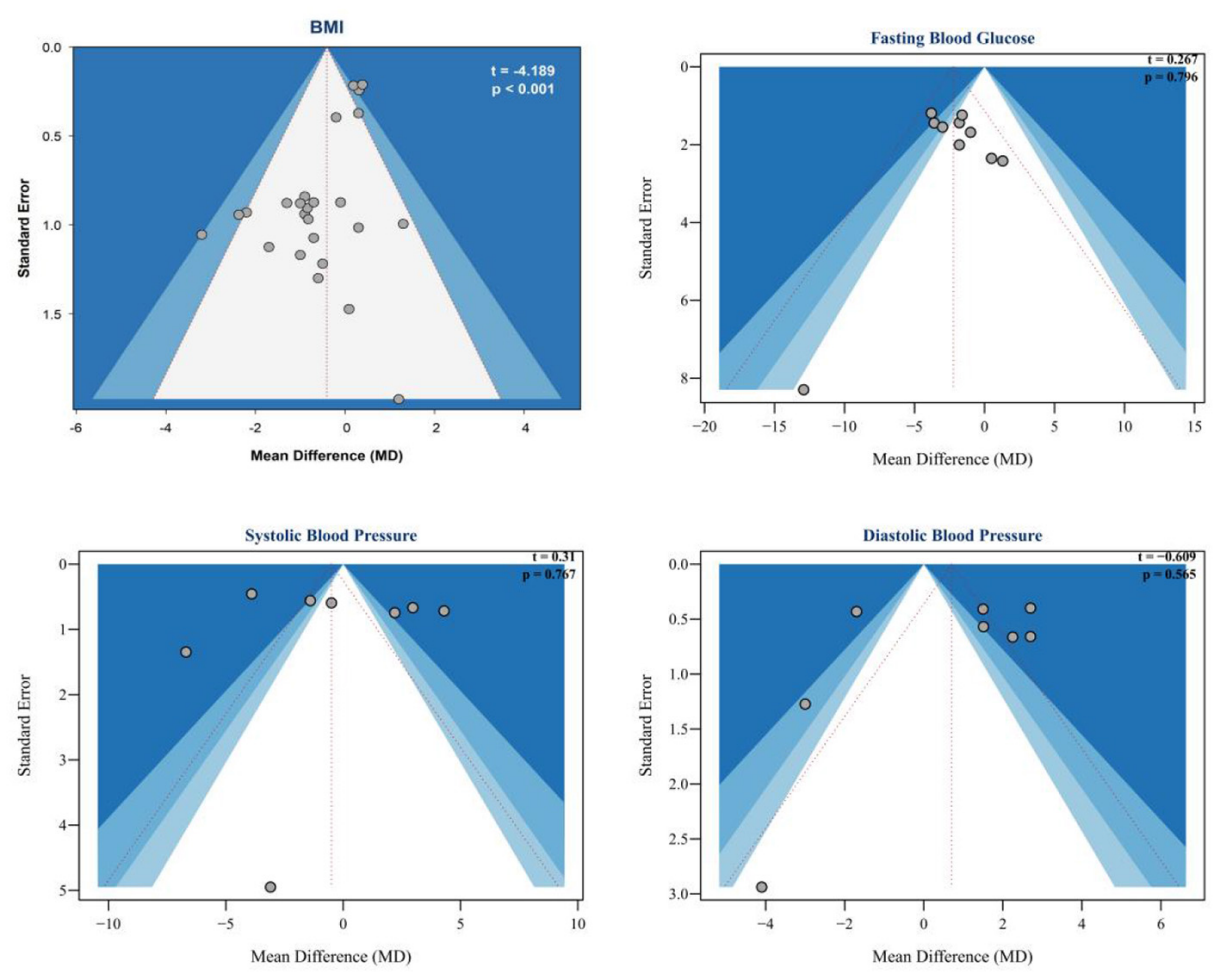
Funnel plot and Egger's publication bias.

**Figure 6:**
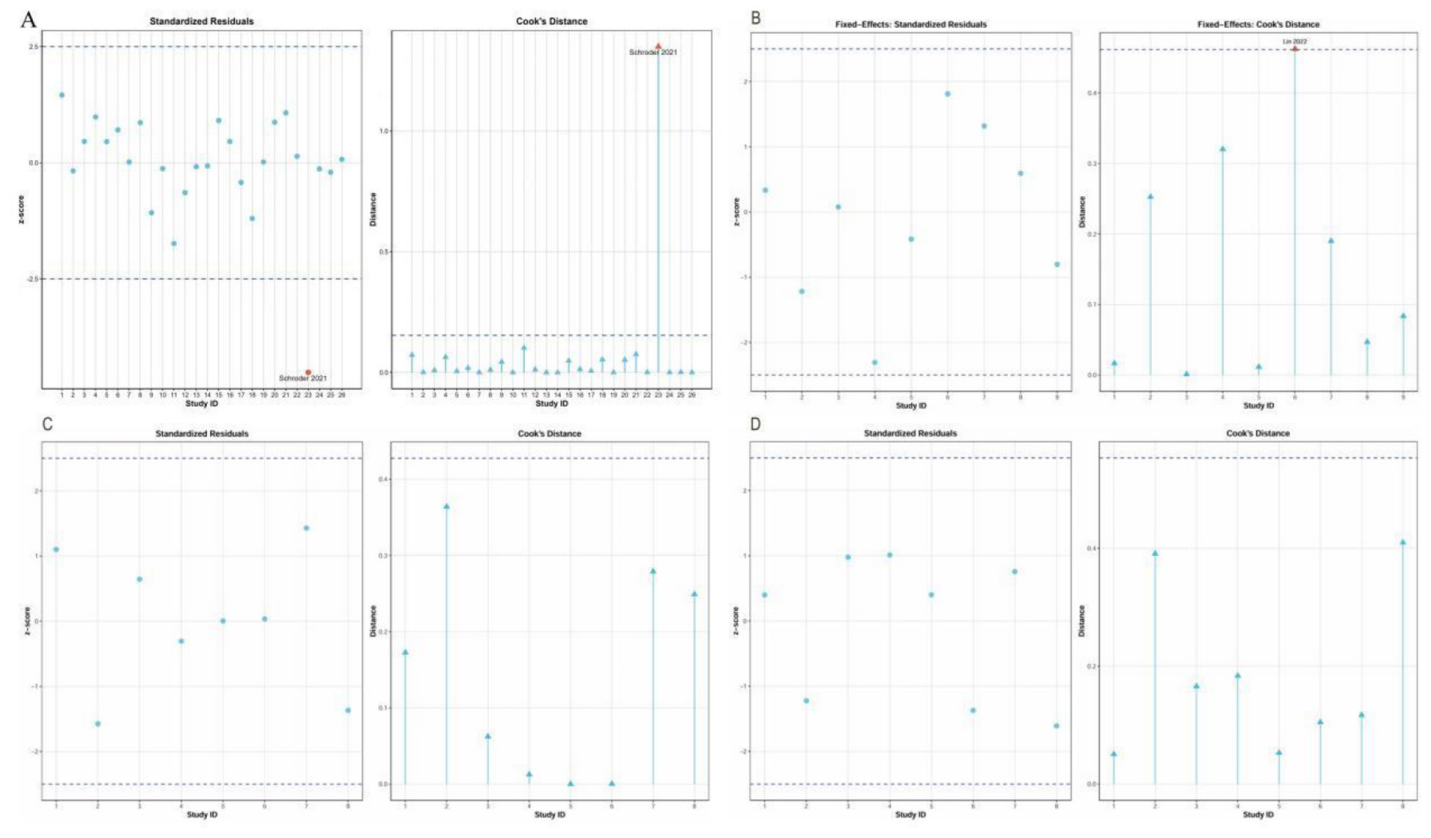
Standardized residuals and Cook's distance threshold plots for model diagnostics. **(A)** BMI under the random-effects model. **(B)** FBG under the fixed-effects model. **(C)** SBP under the random-effects model. **(D)** DBP under the random-effects model. In each panel, the left subplot shows standardized residuals and the right subplot shows Cook's distance by study ID. The horizontal dashed lines indicate the prespecified thresholds used to identify potential outliers or influential studies.

A total of 22 studies involving 1,287 participants were included in the BMI analysis. Substantial heterogeneity was observed (*I*^2^ = 80.2%, *P* < 0.0001); therefore, a random-effects model was applied. The initial pooled analysis showed that IF significantly reduced BMI in women with overweight or obesity (MD = −0.72, 95% CI:−1.25 to−0.20, p = 0.0068). Because the heterogeneity was considerable, influence diagnostics and sensitivity analyses were further conducted. These analyses indicated that Schroder et al. (59) exerted a disproportionate influence on the pooled estimate (see Figure 6A). After exclusion of this study, 25 effect sizes remained in the final BMI analysis. The heterogeneity was reduced to a moderate level (*I*^2^ = 54.3%, *Q* = 41.78, *P* = 0.0137), and the pooled effect remained statistically significant under the random-effects model (MD = −0.41, 95% CI:−0.81 to−0.02, *P* = 0.0396) (see Figure 4A). Publication bias was further assessed using Egger's regression test and the trim-and-fill method. Egger's regression test indicated significant funnel plot asymmetry (*t* = −4.189, *P* < 0.001) (see Figure 5, top left). In addition, the trim-and-fill analysis based on the original model identified no potentially missing studies (k0 = 0), and the pooled estimate remained unchanged after adjustment (MD = −0.72, 95% CI:−1.25 to−0.20). Therefore, the final BMI result presented in the main text and Figure 4A was based on the model after exclusion of the influential study.

FBG Findings: Nine studies involving 531 participants were analyzed, revealing no heterogeneity among them (*I*^2^ = 0%, *P* = 0.5061), which justified the use of a fixed-effects model. The initial combined analysis indicated that IF led to a notable decrease in FBG levels (MD = −2.18, 95% CI:−3.20 to−1.16, *P* < 0.0001) (refer to Figure 4B). Even with the minimal heterogeneity, we conducted a thorough assessment of the results' reliability and potential biases. Egger's regression test did not indicate significant publication bias (*t* = 0.267, *P* = 0.796) (see Figure 5, top right). Analysis of Cook's residuals identified Lin et al. (62) as an outlier (with standardized residuals falling below the minimum threshold and Cook's distance surpassing the limit; see Figure 6B). Sensitivity analysis demonstrated that excluding Lin et al. (62) altered the combined effect size from MD = −2.18 to MD = −2.40, yet the direction and statistical significance of the pooled effect remained unchanged (*P* < 0.0001). Since the main conclusion remained robust even after excluding this outlier, which represented a specific demographic, it was decided to keep the study for the sake of comprehensive reporting. Additionally, a trim-and-fill analysis was conducted to evaluate the impact of bias (see supplementary document). This analysis indicated that two potentially missing studies should be included. After adjustment, the effect size became more pronounced (MD = −2.51, 95% CI:−3.48 to−1.53, *P* < 0.0001), with low heterogeneity still present (*I*^2^ = 15.2%), which supported the robustness of the overall finding.

SBP Findings: Eight studies involving 533 participants were analyzed. The studies exhibited considerable heterogeneity (*I*^2^ = 95.8%; *P* < 0.0001), warranting a random-effects model. The initial analysis indicated that IF did not significantly impact SBP (MD = −0.50, 95% CI:−3.20 to 2.19, *P* = 0.714) (refer to Figure 4C). The high heterogeneity (*I*^2^ = 95.8%, *P* < 0.0001) necessitates careful interpretation of these results. To address the heterogeneity, we conducted a thorough assessment for potential biases and outliers. Egger's regression test did not indicate significant publication bias (*t* = 0.31, *P* = 0.767) (see Figure 5, bottom left). Analysis of Cook's residuals indicated no outlier studies surpassing the threshold (Cook's distance > 3 times the mean), implying that the observed heterogeneity likely arises from variations in study design or measurement rather than from any single outlier (see Figure 6C). Sensitivity analysis demonstrated that excluding any individual study did not yield a statistically significant combined effect size (MD = −1.24 to 0.42, *P* > 0.05). The high level of heterogeneity persisted, reinforcing the conclusion of no significant effect, although the origins of the heterogeneity remain intricate. A trim-and-fill analysis was conducted to assess bias, revealing no need for additional missing studies, and the effect size remained consistent before and after adjustments (see supplementary document). Current evidence indicates that IF does not significantly affect SBP in women with overweight or obesity.

DBP Findings: Eight studies (*n* = 533) were analyzed, revealing considerable heterogeneity (*I*^2^ = 91.5%, *P* < 0.0001), necessitating a random-effects model. The initial aggregated analysis indicated that IF did not significantly impact DBP (MD = 0.71, 95% CI:−0.87 to 2.29, *P* = 0.378) (refer to Figure 4D). The pronounced heterogeneity (*I*^2^ = 91.5%, *P* < 0.0001) raised concerns regarding the reliability of the findings and highlighted the need for further exploration into the causes of this variability. Given the high heterogeneity, we conducted a thorough evaluation of potential biases and outliers. Egger's regression test did not indicate significant publication bias (*t* = −0.609, *P* = 0.565) (see Figure 5, bottom right). Cook's distance metrics were generally low across the studies, with no outliers surpassing the threshold (Cook's distance > 3 times the mean) identified (see Figure 6D). This suggests that the observed high heterogeneity may stem from widespread differences in study design or measurement rather than from any single outlier. Sensitivity analysis indicated that excluding any individual study did not yield a statistically significant combined effect size (MD = 0.37 to 1.23, *P* > 0.05), and the heterogeneity persisted. Significantly, when studies by Haganes (49) or Schroder (73) were excluded, the effect sizes shifted to MD = 1.21 (*P* = 0.105) and MD = 1.23 (*P* = 0.083), respectively, indicating a change in trend, although still not statistically significant, suggesting these studies affected the overall effect direction. A trim-and-fill analysis stated no need to add missing studies, with the effect size remaining consistent before and after adjustments (see supplementary document). Current evidence indicates that IF does not significantly affect DBP in women with overweight or obesity.

### Subgroup analyses

**Figure 7:**
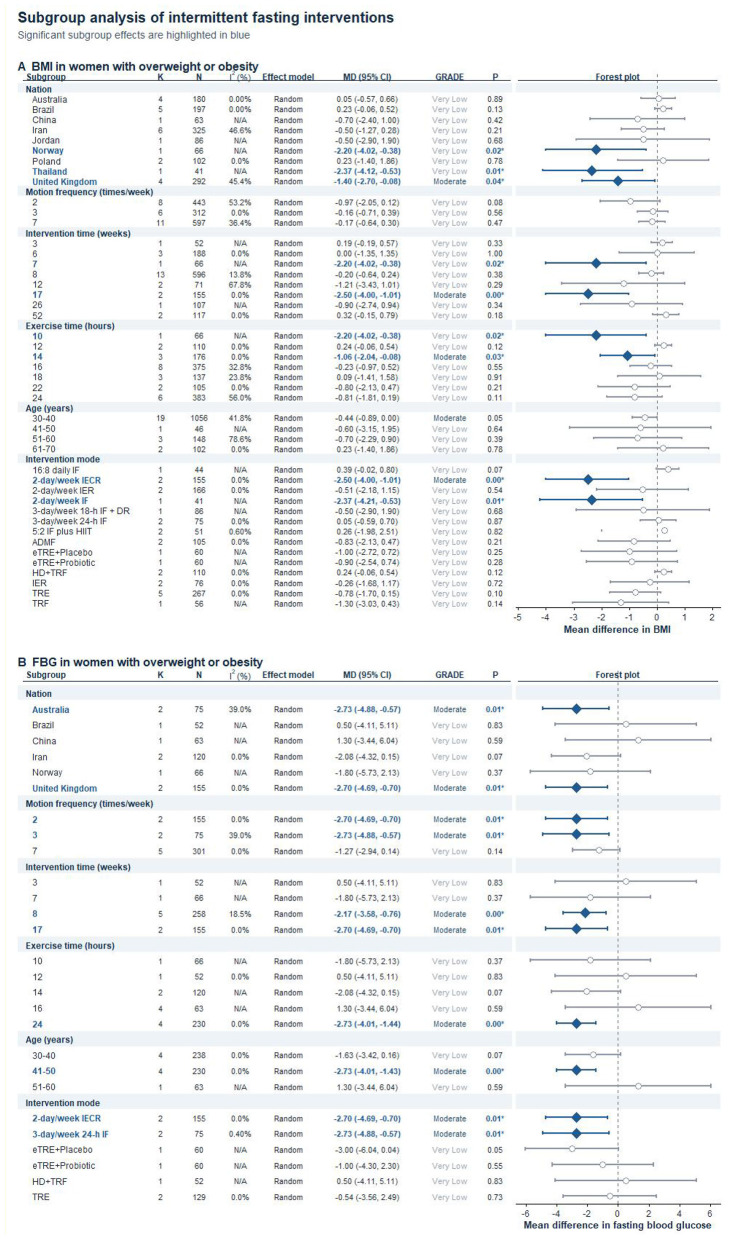
Subgroup analysis of IF interventions on BMI and FBG in women with overweight or obesity.**(A)** Subgroup effects on BMI. **(B)** Subgroup effects on FBG. Effect sizes are presented as mean differences (MDs) with 95% confidence intervals. Subgroups were stratified by nation, intervention frequency, intervention duration, fasting duration per session, age, and intervention mode. Statistically significant subgroup effects are highlighted in blue.

A detailed subgroup analysis was conducted to pinpoint significant elements affecting the impact of IF on BMI and FBG levels among women with overweight or obesity. The variables examined included geographical location, intervention frequency, intervention duration, fasting duration per session, participants' age, and intervention mode. The results from these subgroups were assessed using the GRADE framework to determine the evidence's credibility (63). Further information can be found in Figure 7.

The impact of IF on BMI among women with overweight or obesity showed variation across subgroups. A relatively larger point estimate for BMI reduction was observed in the 24-h subgroup (K = 6, MD = −0.81, *P* = 0.11, GRADE: Very Low), the twice-weekly subgroup (K = 8, MD = −0.97, *P* = 0.08, GRADE: Very Low), and the 17-week subgroup (K = 2, MD = −2.50, *P* = 0.00, GRADE: Moderate). A potentially more responsive pattern was observed in the 30–40-year subgroup (K = 19, MD = −0.44, *P* = 0.05, GRADE: Moderate), whereas the 41–50-year subgroup included only one comparison and was not significant (K = 1, MD = −0.60, *P* = 0.64, GRADE: Very Low). The 30–40-year subgroup contributed the largest number of effect sizes and showed a borderline estimate (MD = −0.44, *P* = 0.05), whereas the estimate for the 41–50-year subgroup was based on only one comparison and was not statistically significant. Intervention-mode sub-grouping also suggested variability. Significant BMI reductions were observed in the 2-day/week IECR subgroup (K = 2, MD = −2.50, 95% CI:−4.00 to−1.01, *P* = 0.00, GRADE: Moderate) and the 2-day/week IF subgroup (K = 1, MD = −2.37, 95% CI:−4.21 to−0.53, *P* = 0.01, GRADE: Very Low), whereas the remaining intervention-mode subgroups were not statistically significant. Geographical variation was also observed, with statistically significant subgroup estimates identified in Norway, Thailand, and the United Kingdom, whereas the subgroup estimates for Australia, Brazil, China, Iran, Jordan, and Poland were not significant. However, these subgroup findings should be interpreted cautiously because several subgroup estimates were based on single studies, most GRADE ratings ranged from low to very low, and heterogeneity remained notable in some strata. Therefore, the subgroup results may be better regarded as exploratory patterns rather than definitive evidence for an optimal regimen. Taken together, the BMI subgroup findings are better regarded as exploratory patterns than as definitive evidence for an optimal intervention regimen.

The impact of IF on FBG levels in women with overweight or obesity also showed variation across subgroups, although the overall certainty of evidence remained limited. A relatively larger reduction in FBG was observed in the 24-h subgroup (K = 4, MD = −2.73, *P* = 0.00, GRADE: Moderate), the twice-weekly subgroup (K = 2, MD = −2.70, *P* = 0.01, GRADE: Moderate), the three-times-weekly subgroup (K = 2, MD = −2.73, *P* = 0.01, GRADE: Moderate), and the 8-week subgroup (K = 5, MD = −2.17, *P* = 0.00, GRADE: Moderate). A potentially more responsive pattern was observed in women aged 41–50 years (K = 4, MD = −2.73, *P* = 0.00, GRADE: Moderate), whereas the 30–40-year subgroup was not significant (K = 4, MD = −1.63, *P* = 0.07, GRADE: Very Low). The 41–50-year subgroup showed a significant effect size with no observed heterogeneity (*I*^2^ = 0.0%), whereas the 30–40-year subgroup did not reach statistical significance. Intervention-mode sub-grouping also showed variability in FBG response. Significant reductions were observed in the 2-day/week IECR subgroup (K = 2, MD = −2.70, 95% CI:−4.69 to−0.70, *P* = 0.01, GRADE: Moderate) and the 3-day/week 24-h IF subgroup (K = 2, MD = −2.73, 95% CI:−4.88 to−0.57, *P* = 0.01, GRADE: Moderate), whereas the remaining intervention-mode subgroups were not statistically significant. At the same time, several subgroup findings remained unstable because many subgroup comparisons were supported by small numbers of studies and the certainty of evidence ranged from moderate to very low. Therefore, these subgroup findings are better understood as exploratory signals rather than firm practice recommendations. Within this context, the subgroup findings for FBG are better interpreted as exploratory signals that may inform future trial design, particularly for the 24-h regimen and for women aged 41–50 years.

### Nonlinear regression and dose-response

**Figure 8:**
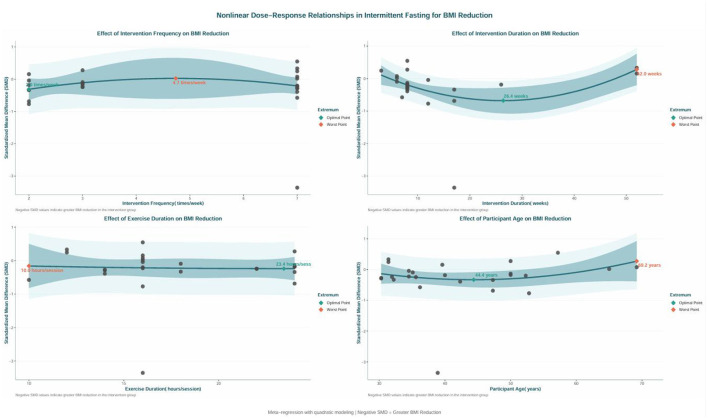
Nonlinear regression plot of the effects of IF on women with overweight or obesity.

**Figure 9:**
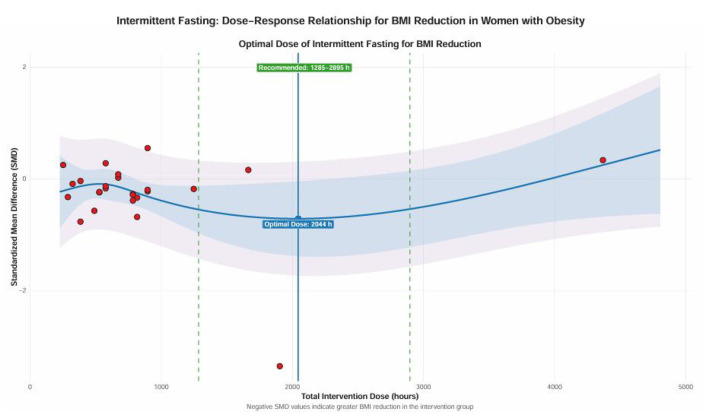
Dose-response relationship plot of the effects of IF on women with overweight or obesity.

In the nonlinear regression and dose–response models (see Figures 8, 9), several key findings emerged: The frequency of intervention appeared to be more favorable when administered approximately twice weekly, with diminishing returns observed as frequency increased, peaking at around 4.7 times weekly; The cumulative impact of the intervention duration appeared greatest at 52 weeks, while the least favorable period was around 26.4 weeks, suggesting that sustained engagement may be more advantageous for lowering BMI; A fasting duration of roughly 24 h appeared to correspond to the most favorable modeled effect, whereas a regimen of 10 h per session yielded the least favorable pattern; Participants around 44.4 years of age appeared to experience greater modeled benefits, while those at 69.2 years showed the least favorable pattern, suggesting that younger to middle-aged individuals may derive greater benefit from the intervention. The comprehensive dose analysis (depicted in Figure 9) indicated that a total intervention time of about 2,044 h was linked to the lowest MD value, with effects remaining fairly consistent between 1,285 and 2,895 h. However, outside this range, the certainty of the modeled efficacy trend became less stable, and the likelihood of a downward trend increased. Due to constraints in data distribution and model calibration, those above the “turning pointsside this range, the certainty of the modeled efficacy trend became less stable, and cross various populations and clinical environments.

## Discussion

The present meta-analysis showed that IF was associated with significant improvements in BMI and FBG in women with overweight or obesity, whereas no significant effects were observed for systolic or diastolic blood pressure. The initial pooled effect for BMI was significant (MD = −0.72, 95% CI:−1.25 to−0.20), although heterogeneity was substantial. For FBG, a smaller but significant reduction was also observed (MD = −2.18, 95% CI:−3.20 to−1.16). In contrast, the pooled effects for systolic and DBP were not statistically significant. Overall, these findings suggest that IF may be more relevant to weight-related and glycemic outcomes than to blood pressure control in this population, although the BMI result in particular should be interpreted cautiously because of substantial between-study heterogeneity and sensitivity to one influential study.

The positive impact of IF on metabolic parameters in women with overweight or obesity can be linked to various physiological processes. This dietary approach enhances insulin sensitivity and stimulates autophagy by activating the AMPK signaling pathway, which, in turn, boosts energy metabolism and facilitates fat oxidation (64). Additionally, it plays a crucial role in modulating the gut microbiota by increasing levels of beneficial bacteria, such as Muribaculaceae and Bacteroides, thereby contributing to reduced FBG levels (65). Furthermore, IF helps align the circadian rhythm, improving blood pressure management and metabolic efficiency. Sleep-related factors may also have influenced the observed metabolic outcomes. Prior quantitative evidence suggests that short sleep is associated with a higher likelihood of obesity in adults (OR = 1.55, 95% CI: 1.43–1.68). In parallel, both the quantity and quality of sleep have been linked to glycemic dysregulation: a systematic review and meta-analysis reported that short sleep (RR = 1.28), long sleep (RR = 1.48), difficulty initiating sleep (RR = 1.57), and difficulty maintaining sleep (RR = 1.84) were each associated with incident type 2 diabetes. Another meta-analysis further showed that short sleep, long sleep, and poor sleep quality were associated with higher HbA1c levels (WMD = 0.23%, 0.13%, and 0.35%, respectively). Therefore, sleep duration and sleep quality should be considered plausible modifiers or confounders when interpreting BMI- and FBG-related responses to IF interventions (66–68). Research by Sutton et al. (12) demonstrated that eTRE positively affected insulin sensitivity and blood pressure without necessitating weight loss (12); similarly, Jamshed et al. (21) validated that the metabolic advantages of IF are somewhat independent of weight fluctuations (21). Taken together, these mechanisms may partly explain why IF was associated with favorable changes in BMI and FBG in the present review, although these interpretations remain constrained by heterogeneity across studies and the limited certainty of the available evidence (69–71).

Analysis of subgroups indicated notable variations in the impact of IF across different scenarios (72). For BMI, relatively larger point estimates were observed in the 24-h fasting-duration subgroup and in the 17-week subgroup, whereas the twice-weekly subgroup showed the largest point estimate in the frequency stratification. For FBG, significant reductions were observed in the 24-h subgroup, in the 8- and 17-week subgroups, and in women aged 41–50 years. When examining demographic factors, the 30–40-year subgroup contributed the largest number of BMI effect sizes and showed a borderline estimate, whereas a significant reduction in FBG was observed in the 41–50-year subgroup. Additionally, geographical variation was observed across subgroup strata. These findings suggest potential variation across intervention characteristics and participant strata, but they do not establish a definitive optimal regimen. A similar exploratory pattern was observed for intervention mode: 2-day/week IECR showed significant subgroup effects for both BMI and FBG, whereas 2-day/week IF was associated with a significant BMI reduction and 3-day/week 24-h IF was associated with a significant FBG reduction. However, most intervention-mode subgroup estimates were based on one or two comparisons and were supported by low or very low certainty evidence, so these findings should not be interpreted as evidence of regimen superiority. One investigation revealed that a regimen of veryh fasting, twice weeklyekting, twice vealed that a regimen of very low cerweeks among women with overweight or obesity (52). Likewise, other research indicated that women with overweight or obesity, averaging 50 years old, experienced substantial improvements in FBG with a therh fasting, three times weeklyeks g,erienced suweeks (41). However, these findings were derived from individual trials and should not be interpreted as definitive evidence for an optimal intervention schedule.

The dose–response analysis provided additional quantitative insight into the association between intervention characteristics and modeled BMI outcomes (37). The nonlinear model suggested that an intervention frequency around twice weekly may be relatively favorable, with diminishing modeled benefit beyond this point. A longer intervention duration appeared to be associated with more favorable modeled effects, with the modeled benefit peak occurring around 52 weeks. The total duration of fasting demonstrated consistent effects across a range of 1,285 to 2,895 h, with around 2,044 h yielding the lowest modeled MD value (35). This finding aligns with the research by Varady et al. (8), which suggests that overly frequent fasting may increase physiological stress, while sustained adherence is essential for achieving cumulative metabolic advantages (8). These dose–response findings should be interpreted as exploratory rather than prescriptive, and they do not establish a definitive optimal intervention schedule. The present findings should not be directly generalized to RIF, because although RIF is a form of IF and has been associated with modest body-weight reduction and selected glucometabolic benefits in prior systematic reviews (28, 29), the published RIF evidence has largely been derived from a different evidence base and population structure than the RCT-focused synthesis used in this review.

This research offered solid evidence for the tailored use of IF among women with overweight or obesity. Nonetheless, subsequent studies should implement RCTs with extended follow-up periods (e.g., beyond 2 years) to confirm the long-term effectiveness of the intervention. Additionally, it is essential to expand sample sizes and incorporate diverse ethnic and cultural groups to enhance the applicability of the findings. Future work should also aim to delineate the specific populations suited to various IF models (e.g., eTRE versus ADF) to improve dose–response parameters, and to incorporate sleep-related variables such as sleep duration, sleep quality, and sleep hygiene into trial design and reporting, so that the independent metabolic effects of IF can be interpreted more precisely (31).

### Limitations

Several limitations should be acknowledged. First, most included trials were short-term, and some outcomes–particularly FBG and blood pressure–were supported by relatively few studies with small sample sizes, which limits estimate precision and the assessment of long-term effects. Second, substantial heterogeneity was observed in several analyses, and the included trials varied in fasting protocols, comparator conditions, participant age, intervention duration, and possible co-interventions such as exercise; these factors may have introduced residual confounding that could not be fully addressed using aggregate study-level data. Third, sleep-related variables, including sleep duration, sleep quality, and sleep hygiene, were not prespecified for quantitative synthesis in the present review. Although a small number of included trials reported selected sleep-related outcomes, these measures were heterogeneous and insufficiently comparable across studies, which prevented formal quantitative evaluation of their potential confounding effects on BMI and FBG outcomes. Therefore, part of the observed metabolic effects attributed to IF may have been influenced by unmeasured or inconsistently measured sleep-related factors. Fourth, this review focused on prespecified clinically relevant outcomes (BMI, FBG, and blood pressure) and did not quantitatively synthesize other anthropometric or metabolic indicators, such as waist circumference, body fat percentage, fasting insulin, or HOMA-IR. Therefore, the findings should not be interpreted as a comprehensive assessment of all metabolic effects of IF in women with overweight or obesity. Finally, only English-language full-text studies were included, which may have introduced language bias by excluding relevant studies published in other languages, particularly those reporting null or less favorable findings. This bias may not be fully captured by conventional publication-bias diagnostics, and many subgroup findings were supported by limited or low-certainty evidence; accordingly, the overall findings should be interpreted with caution.

## Conclusions

IF may improve BMI and FBG in women with overweight or obesity, but no significant effects were observed for blood pressure outcomes in the present analysis. The dose–response findings may suggest potentially favorable intervention patterns, but these results should be considered exploratory rather than prescriptive because of heterogeneity across studies, variation in intervention and comparator conditions, the limited evidence base for some outcomes, and the possibility of language bias related to the English-only eligibility criteria. Future well-designed trials with longer follow-up and more homogeneous intervention reporting are needed to confirm these findings and refine practical recommendations.

## Data Availability

The raw data supporting the conclusions of this article will be made available by the authors, without undue reservation.

## References

[1] Naous E Achkar A Mitri J. Intermittent fasting and its effects on weight, glycemia, lipids, and blood pressure: a narrative review. Nutrients. (2023) 15:3661. doi: 10.3390/nu15163661 37630851 PMC10459308

[2] Ren X Jiang M Han L Zheng X. Estimated glucose disposal rate and risk of cardiovascular disease: evidence from the China Health and Retirement Longitudinal Study. BMC Geriatr. (2022) 22:968. doi: 10.1186/s12877-022-03689-x 36517754 PMC9753298

[3] Cho Y Hong N Kim K Cho S Lee M Lee Y . The effectiveness of intermittent fasting to reduce body mass index and glucose metabolism: a systematic review and meta-analysis. JCM. (2019) 8:1645. doi: 10.3390/jcm8101645 31601019 PMC6832593

[4] De Cabo R Mattson MP. Effects of intermittent fasting on health, aging, and disease. N Engl J Med. (2019) 381:2541–51. doi: 10.1056/NEJMra1905136 31881139

[5] Headland ML Clifton PM Keogh JB. Impact of intermittent vs. continuous energy restriction on weight and cardiometabolic factors: a 12-month follow-up. Int J Obes. (2020) 44:1236–42. doi: 10.1038/s41366-020-0525-7 31937907

[6] Liu H Liu S Wang K Zhang T Yin L Liang J . Time-dependent effects of physical activity on cardiovascular risk factors in adults: a systematic review. Int J Environ Res Public Health. (2022) 19:14194. doi: 10.3390/ijerph192114194 36361072 PMC9655086

[7] Castelnuovo G Pietrabissa G Manzoni GM Cattivelli R Rossi A Novelli M . Cognitive behavioral therapy to aid weight loss in obese patients: current perspectives. PRBM. (2017) 10:165–73. doi: 10.2147/PRBM.S113278 28652832 PMC5476722

[8] Varady KA Cienfuegos S Ezpeleta M Gabel K. Clinical application of intermittent fasting for weight loss: progress and future directions. Nat Rev Endocrinol. (2022) 18:309–21. doi: 10.1038/s41574-022-00638-x 35194176

[9] Patterson RE Sears DD. Metabolic Effects of Intermittent Fasting. Annu Rev Nutr. (2017) 37:371–93. doi: 10.1146/annurev-nutr-071816-064634 28715993 PMC13170603

[10] Grundler F Mesnage R Michalsen A Wilhelmi De Toledo F. Blood Pressure Changes in 1610 Subjects With and Without Antihypertensive Medication During Long-Term Fasting. JAHA. (2020) 9:e018649. doi: 10.1161/JAHA.120.018649 33222606 PMC7763762

[11] Wang W Wei R Pan Q Guo L. Beneficial effect of time-restricted eating on blood pressure: a systematic meta-analysis and meta-regression analysis. Nutr Metab. (2022) 19:77. doi: 10.1186/s12986-022-00711-2 36348493 PMC9644535

[12] Sutton EF Beyl R Early KS Cefalu WT Ravussin E Peterson CM. Early Time-Restricted Feeding Improves Insulin Sensitivity, Blood Pressure, and Oxidative Stress Even without Weight Loss in Men with Prediabetes. Cell Metabolism (2018) 27:1212-1221.e3. doi: 10.1016/j.cmet.2018.04.010 29754952 PMC5990470

[13] Ravussin E Beyl RA Poggiogalle E Hsia DS Peterson CM. Early time-restricted feeding reduces appetite and increases fat oxidation but does not affect energy expenditure in humans. Obesity. (2019) 27:1244–54. doi: 10.1002/oby.22518 31339000 PMC6658129

[14] Gabel K Hoddy KK Haggerty N Song J Kroeger CM Trepanowski JF . Effects of 8-h time restricted feeding on body weight and metabolic disease risk factors in obese adults: a pilot study. Nutrition and Healthy Aging. (2018) 4:345–53. doi: 10.3233/NHA-170036 29951594 PMC6004924

[15] Liu D Huang Y Huang C Yang S Wei X Zhang P . Calorie Restriction with or without Time-Restricted Eating in Weight Loss. N Engl J Med. (2022) 386:1495–504. doi: 10.1056/NEJMoa2114833 35443107

[16] Haider S. The Metabolic Triad-intermittent Fasting as a Shared Therapeutic Avenue for Non-alcoholic Fatty Liver Disease, Type 2 Diabetes Mellitus, and Gastroesophageal Reflux Disease. J Med Res. (2025) 11:101–2. doi: 10.4103/JMR.JMR_42_25

[17] Cermáková E Forejt M Cermák M. The influence of intermittent fasting on selected human anthropometric parameters. Int J Med Sci. (2024) 21:2630–9. doi: 10.7150/ijms.99116 39512696 PMC11539393

[18] Couto S Cenit MC Montero J Iguacel I. The impact of intermittent fasting and Mediterranean diet on older adults' physical health and quality of life: A randomized clinical trial. Nutr Metab Cardiovasc Dis. (2025) 35:104132. doi: 10.1016/j.numecd.2025.104132 40451678

[19] Zhang S Sun B Sun L Zou S Chen Q. Effect of intermittent fasting on obesity and metabolic indices in patients with metabolic syndrome: a systematic review and meta analysis. BMC Endocr Disord. (2025) 25:130. doi: 10.1186/s12902-025-01952-x 40369509 PMC12076832

[20] Wilkinson MJ Manoogian ENC Zadourian A Lo H Fakhouri S Shoghi A . Ten-Hour Time-Restricted Eating Reduces Weight, Blood Pressure, and Atherogenic Lipids in Patients with Metabolic Syndrome. Cell Metabolism (2020) 31:92-104.e5. doi: 10.1016/j.cmet.2019.11.004 31813824 PMC6953486

[21] Jamshed H Steger FL Bryan DR Richman JS Warriner AH Hanick CJ . Effectiveness of early time-restricted eating for weight loss, fat loss, and cardiometabolic health in adults with obesity: a randomized clinical trial. JAMA Intern Med. (2022) 182:953. doi: 10.1001/jamainternmed.2022.3050 35939311 PMC9361187

[22] Chung H Chou W Sears DD Patterson RE Webster NJG Ellies LG. Time-restricted feeding improves insulin resistance and hepatic steatosis in a mouse model of postmenopausal obesity. Metabolism. (2016) 65:1743–54. doi: 10.1016/j.metabol.2016.09.006 27832862 PMC5123758

[23] Roky R Aadil N Krami AM Benaji B Errabih I Abdelrahim DN . Sex as a Biological Factor in the Changes in Disease Patients During Ramadan Intermittent Fasting: A Systematic Review. Front Nutr (2022) 9:908674. doi: 10.3389/fnut.2022.908674 35845800 PMC9284209

[24] Abdelrahim DN Roky R Krami AM Aadil N Faris ME. Sex as a biological determinant in anthropometric, biochemical, and dietary changes during Ramadan intermittent fasting in healthy people: A systematic review. Diabetes Metab Syndr. (2023) 17:102762. doi: 10.1016/j.dsx.2023.102762 37141819

[25] Sun M-L Yao W Wang X-Y Gao S Varady KA Forslund SK . Intermittent fasting and health outcomes: an umbrella review of systematic reviews and meta-analyses of randomised controlled trials. eClinicalMedicine. (2024) 70:102519. doi: 10.1016/j.eclinm.2024.102519 38500840 PMC10945168

[26] Mohamed YA Abouelmagd M Elbialy A Elwassefy M Kyrillos F. Effect of intermittent fasting on lipid biokinetics in obese and overweight patients with type 2 diabetes mellitus: prospective observational study. Diabetol Metab Syndr. (2024) 16:4. doi: 10.1186/s13098-023-01234-3 38172970 PMC10763162

[27] Yuan X Wang J Yang S Gao M Cao L Li X . Effect of intermittent fasting diet on glucose and lipid metabolism and insulin resistance in patients with impaired glucose and lipid metabolism: a systematic review and meta-analysis. Int J Endocrinol. (2022) 2022:1–9. doi: 10.1155/2022/6999907 35371260 PMC8970877

[28] Jahrami HA Alsibai J Clark CCT Faris MA-IE. A systematic review, meta-analysis, and meta-regression of the impact of diurnal intermittent fasting during Ramadan on body weight in healthy subjects aged 16 years and above. Eur J Nutr. (2020) 59:2291–316. doi: 10.1007/s00394-020-02216-1 32157368

[29] Faris MA Jahrami H BaHammam A Kalaji Z Madkour M Hassanein M. Systematic review, meta-analysis, and meta-regression of the impact of diurnal intermittent fasting during Ramadan on glucometabolic markers in healthy subjects. Diabetes Res Clin Pract. (2020) 165:108226. doi: 10.1016/j.diabres.2020.108226 32446800

[30] Ranneh Y Hamsho M Shkorfu W Terzi M Fadel A. Effect of intermittent fasting on anthropometric measurements, metabolic profile, and hormones in women with polycystic ovary syndrome: a systematic review and meta-analysis. Nutrients. (2025) 17:2436. doi: 10.3390/nu17152436 40806019 PMC12348862

[31] Pavlou V Cienfuegos S Lin S Ezpeleta M Ready K Corapi S . Effect of time-restricted eating on weight loss in adults with type 2 diabetes: a randomized clinical trial. JAMA Netw Open. (2023) 6:e2339337. doi: 10.1001/jamanetworkopen.2023.39337 37889487 PMC10611992

[32] PRISMA-P Group Moher D Shamseer L Clarke M Ghersi D Liberati A . Preferred reporting items for systematic review and meta-analysis protocols (PRISMA-P) 2015 statement. Syst Rev. (2015) 4:1. doi: 10.1186/2046-4053-4-1 25554246 PMC4320440

[33] Khan S. Meta-Analysis: Methods for Health and Experimental Studies. Singapore: Springer Singapore. (2020).

[34] Lau J Ioannidis JPA Schmid CH. Quantitative Synthesis in Systematic Reviews. Ann Intern Med. (1997) 127:820–6. doi: 10.7326/0003-4819-127-9-199711010-00008 9382404

[35] Chuan Hong Jing Zhang Yang Li Elena Elia Richard Riley Yong Chen. A regression-based method for detecting publication bias in multivariate meta-analysis. Res Synth Methods. (2020) 11:364–376. doi: 10.48550/arXiv.2002.04775

[36] Viechtbauer W Cheung MW-L. Outlier and influence diagnostics for meta-analysis. Res Synth Method. (2010) 1:112–25. doi: 10.1002/jrsm.11 26061377

[37] Van Houwelingen HC Arends LR Stijnen T. Advanced methods in meta-analysis: multivariate approach and meta-regression. Stat Med. (2002) 21:589–624. doi: 10.1002/sim.1040 11836738

[38] Larry V. Hedges. Statistical Methods for Meta-Analysis. (1982). 79 p. Available online at: https://sc.panda985.com/extdomains/books.google.com/books/about/Statistical_Methods_for_Meta_Analysis.html?hl=zh-CN&id=7GviBQAAQBAJ [Accessed September 22, 2025]

[39] Sterne JAC Savović J Page MJ Elbers RG Blencowe NS Boutron I . RoB 2: a revised tool for assessing risk of bias in randomised trials. BMJ. (2019) 366:l4898. doi: 10.1136/bmj.l4898 31462531

[40] Schünemann H Brożek J Guyatt G. GRADE handbook for grading quality of evidence and strength of recommendations. GRADE Working Group. (2013).

[41] Hutchison AT Liu B Wood RE Vincent AD Thompson CH O'Callaghan NJ . Effects of Intermittent Versus Continuous Energy Intakes on Insulin Sensitivity and Metabolic Risk in Women with Overweight. Obesity. (2019) 27:50–8. doi: 10.1002/oby.22345 30569640

[42] Abu Salma BM Thekrallah F Qatawneh A Hasan H Shawaqfeh S Altarawneh M. Effect of intermittent fasting on improve body composition and anthropometric measurements of women with polycystic ovarian syndrome. Nutr Clin Diet Hosp. (2024) 44:122–9. doi: 10.12873/442abu

[43] Fagundes GBP Tibães JRB Silva ML Braga MM Silveira ALM Teixeira AL . Metabolic and behavioral effects of time-restricted eating in women with overweight or obesity: Preliminary findings from a randomized study. Nutrition (2023) 107:111909. doi: 10.1016/j.nut.2022.111909 36571891

[44] Batitucci G Faria Junior EV Nogueira JE Brandão CFC Abud GF Ortiz GU. Impact of Intermittent Fasting Combined With High-Intensity Interval Training on Body Composition, Metabolic Biomarkers, and Physical Fitness in Women With Obesity. Front Nutr. (2022) 9:884305. doi: 10.3389/fnut.2022.884305 35694163 PMC9178202

[45] Irani H Abiri B Khodami B Yari Z Lafzi Ghazi M Hosseinzadeh N . Effect of time restricted feeding on anthropometric measures, eating behavior, stress, serum levels of BDNF and LBP in overweight/obese women with food addiction: a randomized clinical trial. Nutr Neurosci. (2024) 27:577–589. doi: 10.1080/1028415X.2023.2234704 37436939

[46] Harvie M Wright C Pegington M McMullan D Mitchell E Martin B . The effect of intermittent energy and carbohydrate restriction *v*. daily energy restriction on weight loss and metabolic disease risk markers in overweight women. Br J Nutr. (2013) 110:1534–47. doi: 10.1017/S0007114513000792 23591120 PMC5857384

[47] Pureza IROM Melo ISV Macena ML Praxedes DRS Vasconcelos LGL Silva-Júnior AE . Acute effects of time-restricted feeding in low-income women with obesity placed on hypoenergetic diets: Randomized trial. Nutrition. (2020) 77:110796. doi: 10.1016/j.nut.2020.110796 32428840

[48] de Oliveira Maranhão Pureza IR da Silva Junior AE Silva Praxedes DR Lessa Vasconcelos LG de Lima Macena M Vieira de Melo IS . Effects of time-restricted feeding on body weight, body composition and vital signs in low-income women with obesity: A 12-month randomized clinical trial. Clinical Nutrition. (2021) 40:759–66. doi: 10.1016/j.clnu.2020.06.036 32713721

[49] Haganes KL Silva CP Eyjólfsdóttir SK Steen S Grindberg M Lydersen S. Time-restricted eating and exercise training improve HbA1c and body composition in women with overweight/obesity: A randomized controlled trial. Cell Metabolism. (2022) 34:1457-1471.e4. doi: 10.1016/j.cmet.2022.09.003 36198292

[50] Beaulieu K Casanova N Oustric P Turicchi J Gibbons C Hopkins M . Matched Weight Loss Through Intermittent or Continuous Energy Restriction Does Not Lead To Compensatory Increases in Appetite and Eating Behavior in a Randomized Controlled Trial in Women with Overweight and Obesity. J Nutrition. (2020) 150:623–633. doi: 10.1093/jn/nxz296 31825067

[51] Gray KL Clifton PM Keogh JB. The effect of intermittent energy restriction on weight loss and diabetes risk markers in women with a history of gestational diabetes: a 12-month randomized control trial. Am J Clin Nutr. (2021) 114:794–803. doi: 10.1093/ajcn/nqab058 33831950

[52] Harvie MN Pegington M Evans G Howell A Mattson MP Martin B . The effects of intermittent or continuous energy restriction on weight loss and metabolic disease risk markers: a randomized trial in young overweight women. Int J Obes. (2011) 35:714–727. doi: 10.1038/ijo.2010.171 20921964 PMC3017674

[53] Ranjbar M Shab-Bidar S Rostamian A Mohammadi H Tavakoli A Djafarian K. Effects of intermittent fasting diet in overweight and obese postmenopausal women with rheumatoid arthritis: A randomized controlled clinical trial. Complement Ther Med. (2025) 91:103189. doi: 10.1016/j.ctim.2025.103189 40354829

[54] Domaszewski P Konieczny M Pakosz P Baczkowicz D Sadowska-Krepa E. Effect of a Six-Week Intermittent Fasting Intervention Program on the Composition of the Human Body in Women over 60 Years of Age. IJERPH. (2020) 17:4138. doi: 10.3390/ijerph17114138 32531956 PMC7312819

[55] Domaszewski P Konieczny M Dybek T Łukaniszyn-Domaszewska K Anton S Sadowska-Krepa E . Comparison of the effects of six-week time-restricted eating on weight loss, body composition, and visceral fat in overweight older men and women. Experimental Gerontology. (2023) 174:112116. doi: 10.1016/j.exger.2023.112116 36739795

[56] Keawtep P Sungkarat S Boripuntakul S Sa-nguanmoo P Wichayanrat W Chattipakorn SC . Effects of combined dietary intervention and physical-cognitive exercise on cognitive function and cardiometabolic health of postmenopausal women with obesity: a randomized controlled trial. Int J Behav Nutr Phys Act. (2024) 21:28. doi: 10.1186/s12966-024-01580-z 38443944 PMC10913568

[57] Hooshiar SH Yazdani A Jafarnejad S. Alternate-day modified fasting diet improves weight loss, subjective sleep quality and daytime dysfunction in women with obesity or overweight: a randomized, controlled trial. Front Nutr. (2023) 10:1174293. doi: 10.3389/fnut.2023.1174293 37275639 PMC10233006

[58] Hooshiar SH Yazdani A Jafarnejad S. Does an alternate-day modified fasting diet improve premenstrual syndrome symptoms and health-related quality of life in obese or overweight women with premenstrual syndrome? A randomized, controlled trial. Front Nutr. (2024) 10:1298831. doi: 10.3389/fnut.2023.1298831 38268675 PMC10806082

[59] Schroder JD Falqueto H Mânica A Zanini D de Oliveira T de Sá CA . Effects of time-restricted feeding in weight loss, metabolic syndrome and cardiovascular risk in obese women. J Transl Med. (2021) 19:3. doi: 10.1186/s12967-020-02687-0 33407612 PMC7786967

[60] Talebi S Shab-Bidar S Moini A Mohammadi H Djafarian K. The effects of time-restricted eating alone or in combination with probiotic supplementation in comparison with a calorie-restricted diet on endocrine and metabolic profiles in women with polycystic ovary syndrome: a randomized clinical trial. Diabetes Obesity Metabolism. (2024) 26:4468–79. doi: 10.1111/dom.15801 39143654

[61] Teong XT Hutchison AT Liu B Wittert GA Lange K Banks S . Eight weeks of intermittent fasting versus calorie restriction does not alter eating behaviors, mood, sleep quality, quality of life and cognitive performance in women with overweight. Nutrition Research. (2021) 92:32–9. doi: 10.1016/j.nutres.2021.06.006 34274552

[62] Lin YJ Wang YT Chan LC Chu NF. Effect of time-restricted feeding on body composition and cardio-metabolic risk in middle-aged women in Taiwan. Nutrition. (2022) 93:111504. doi: 10.1016/j.nut.2021.111504 34763309

[63] Guyatt GH Oxman AD Vist GE Kunz R Falck-Ytter Y Alonso-Coello P . an emerging consensus on rating quality of evidence and strength of recommendations. BMJ. (2008) 336:924–6. doi: 10.1136/bmj.39489.470347.AD 18436948 PMC2335261

[64] Delrue C Speeckaert R Speeckaert MM. The role of intermittent fasting and ketogenic diet in metabolic syndrome and type 2 diabetes. Acta Clin Belg. (2025) 80:100–14. doi: 10.1080/17843286.2025.2540287 40737109

[65] Li Z Chen S Yin B Wei J Wang D Zhou H . Intermittent fasting regulates gut microbiota and serum metabolome profiles in middle-aged mice fed high-fat diet. Nutr Metab. (2025) 22:16. doi: 10.1186/s12986-025-00904-5 40001132 PMC11863773

[66] Cappuccio FP Taggart FM Kandala NB Currie A Peile E Stranges S . Meta-analysis of short sleep duration and obesity in children and adults. Sleep. (2008) 31:619–26. doi: 10.1093/sleep/31.5.619 18517032 PMC2398753

[67] Cappuccio FP D'Elia L Strazzullo P Miller MA. Quantity and Quality of Sleep and Incidence of Type 2 DiabetesA systematic review and meta-analysis. Diabetes Care. (2010) 33:414–20. doi: 10.2337/dc09-1124 19910503 PMC2809295

[68] Lee SWH Ng KY Chin WK. The impact of sleep amount and sleep quality on glycemic control in type 2 diabetes: A systematic review and meta-analysis. Sleep Med Rev. (2017) 31:91–101. doi: 10.1016/j.smrv.2016.02.001 26944909

[69] Mazurek J Stachowicz H Adamczyk M Baran A Bartos W Ptasznik M . Intermittent fasting - a diet to fight type 2 diabetes and obesity - current state of knowledge. J Educ Health Sport. (2024) 67:55044. doi: 10.12775/JEHS.2024.67.55044

[70] Matías-Pérez D Hernández-Bautista E García-Montalvo IA. Intermittent fasting may optimize intestinal microbiota, adipocyte status and metabolic health. Asia Pac J Clin Nutr. (2022) 31 (1):16–23. doi: 10.6133/apjcn.202203_31(1).0002 35357099

[71] Ribas-Latre A Fernández-Veledo S Vendrell J. Time-restricted eating, the clock ticking behind the scenes. Front Pharmacol. (2024) 15:1428601. doi: 10.3389/fphar.2024.1428601 39175542 PMC11338815

[72] Lesan V Odaie V Munteanu C. The Role of Different Risk Groups and Treatment Assignment Probabilities in Subgroup Analysis of Randomized Trials. Hematol Oncol. (2025) 43:e70132. doi: 10.1002/hon.70132 40817584

[73] Schroder JD Falqueto H Mânica A Zanini D de Oliveira T de Sá CA . Effects of time-restricted feeding in weight loss, metabolic syndrome and cardiovascular risk in obese women. J Transl Med. (2021) 19:3. doi: 10.1186/s12967-020-02687-0 33407612 PMC7786967

