# Supplementary material for: The effects of intermittent fasting on BMI, fasting blood glucose, and blood pressure in women with overweight or obesity: a systematic review and meta-analysis with dose–response relationships
Source: Front Nutr. 2026 May 14;13:1818813. doi: 10.3389/fnut.2026.1818813 (PMC13218085; doi:10.3389/fnut.2026.1818813)
Supplement: Supplementary file 1 [file Data_Sheet_1.docx]

Table of Contents

[Search Strategy 1](#_Toc12248)

[Funnel plot: IF on BMI in women with overweight or obesity 4](#_Toc510)

[Sensitivity analysis: IF and BMI 4](#_Toc13051)

[Funnel plot: IF on fasting blood glucose in women with overweight or obesity 5](#_Toc23016)

[Sensitivity analysis: IF and fasting blood glucose 5](#_Toc26340)

[Funnel plot: IF on systolic blood pressure in women with overweight or obesity 6](#_Toc27742)

[Sensitivity analysis: IF and systolic blood pressure 6](#_Toc405)

[Funnel plot: IF on diastolic blood pressure in women with overweight or obesity 7](#_Toc4141)

[Sensitivity analysis: IF and diastolic blood pressure 7](#_Toc31772)

[Supplementary Table S1. Reasons for records removed for other documented reasons before title/abstract screening (n = 157) 8](#_Toc10715)

[Supplementary Table S2. Full-text reports excluded with reasons (n = 3969) 8](#_Toc25063)

# Search Strategy

**1. PubMed**

("Intermittent Fasting"[Mesh] OR "Intermittent Fasting"[tiab] OR "Time-Restricted Feeding"[tiab] OR "Time-Restricted Eating"[tiab] OR "Early Time-Restricted Feeding"[tiab] OR eTRF[tiab] OR TRE[tiab] OR TRF[tiab] OR "Alternate-Day Fasting"[tiab] OR "Alternate Day Fasting"[tiab] OR ADF[tiab] OR "Alternate-Day Modified Fasting"[tiab] OR ADMF[tiab] OR "Intermittent Energy Restriction"[tiab] OR IER[tiab] OR "5:2"[tiab] OR "5 2"[tiab] OR "Extended Overnight Fasting"[tiab] OR "Overnight Fasting"[tiab] OR "12-hour fasting"[tiab] OR Fasting[tiab])

AND

(("Obesity"[Mesh] OR obes*[tiab] OR overweight[tiab] OR "Body Mass Index"[tiab] OR BMI[tiab])

AND

("Women"[Mesh] OR women[tiab] OR woman[tiab] OR female*[tiab]))

AND

(("Body Mass Index"[tiab] OR BMI[tiab]) OR ("Fasting Blood Glucose"[Mesh] OR "Fasting Glucose"[tiab] OR "Blood Glucose"[tiab] OR FBG[tiab]) OR ("Blood Pressure"[Mesh] OR "Blood Pressure"[tiab] OR hypertension[tiab] OR BP[tiab]))

AND

("Randomized Controlled Trial"[pt] OR "RCT"[tiab] OR "randomized controlled trial"[tiab] OR randomized[tiab])

NOT

("Review"[pt] OR "Case Reports"[pt] OR "Comment"[pt] OR "Editorial"[pt])

Filters: English, Publication date to 2025/08/01

**2. Cochrane Library**

#1 [mh "Intermittent Fasting"] OR [mh Fasting] OR "intermittent fasting":ti,ab,kw OR "time-restricted feeding":ti,ab,kw OR "time-restricted eating":ti,ab,kw OR "early time-restricted feeding":ti,ab,kw OR eTRF:ti,ab,kw OR TRE:ti,ab,kw OR TRF:ti,ab,kw OR "alternate-day fasting":ti,ab,kw OR "alternate day fasting":ti,ab,kw OR ADF:ti,ab,kw OR "alternate-day modified fasting":ti,ab,kw OR ADMF:ti,ab,kw OR "intermittent energy restriction":ti,ab,kw OR IER:ti,ab,kw OR "5:2":ti,ab,kw OR "5 2":ti,ab,kw OR "extended overnight fasting":ti,ab,kw OR "overnight fasting":ti,ab,kw OR "12-hour fasting":ti,ab,kw

#2 [mh Obesity] OR obesity:ti,ab,kw OR overweight:ti,ab,kw OR "body mass index":ti,ab,kw OR BMI:ti,ab,kw

#3 [mh Women] OR women:ti,ab,kw OR woman:ti,ab,kw OR female:ti,ab,kw

#4 #2 AND #3

#5 [mh "Randomized Controlled Trials"] OR randomized:ti,ab,kw OR "randomized controlled trial":ti,ab,kw OR RCT:ti,ab,kw OR trial:ti,ab,kw

#6 "body mass index":ti,ab,kw OR BMI:ti,ab,kw OR "blood glucose":ti,ab,kw OR "fasting glucose":ti,ab,kw OR "blood pressure":ti,ab,kw OR hypertension:ti,ab,kw

#7 #1 AND #4 AND #5 AND #6

Publication Date to 1 August 2025, Language: English

**3. Web of Science**

TS=("intermittent fasting" OR "time-restricted feeding" OR "time-restricted eating" OR "early time-restricted feeding" OR eTRF OR TRE OR TRF OR "alternate-day fasting" OR "alternate day fasting" OR ADF OR "alternate-day modified fasting" OR ADMF OR "intermittent energy restriction" OR IER OR "5:2" OR "5 2" OR "extended overnight fasting" OR "overnight fasting" OR "12-hour fasting" OR fasting)

AND

TS=("obesity" OR "overweight" OR "body mass index" OR "BMI")

AND

TS=("women" OR "woman" OR "female")

AND

TS=("body mass index" OR BMI OR "fasting blood glucose" OR "fasting glucose" OR "blood glucose" OR "FBG" OR "blood pressure" OR "hypertension" OR "BP")

AND

TS=("randomized controlled trial" OR "RCT" OR "randomized" OR "controlled clinical trial" OR "clinical trial")

NOT

TS=("review" OR "case report" OR "commentary" OR "editorial")

Refined by: [LANGUAGE]: (English) AND [DOCUMENT TYPES]: (ARTICLE)

Timespan: All years to 2025-08-01

**4. PsycINFO**

(fasting OR "intermittent fasting" OR "time-restricted feeding" OR "time-restricted eating" OR "early time-restricted feeding" OR eTRF OR TRE OR TRF OR "alternate-day fasting" OR "alternate day fasting" OR ADF OR "alternate-day modified fasting" OR ADMF OR "intermittent energy restriction" OR IER OR "5:2" OR "5 2" OR "extended overnight fasting" OR "overnight fasting" OR "12-hour fasting" OR dieting OR "eating pattern*" OR "meal timing")

AND

(women OR woman OR female)

AND

(obesity OR overweight OR "body mass index" OR BMI OR adiposity OR "body weight" OR "weight loss" OR "weight management" OR "body composition")

AND

(BMI OR "body mass index" OR glycem OR glucose OR "blood glucose" OR "fasting glucose" OR "fasting blood glucose" OR FBG OR "blood pressure" OR hypertension OR BP OR systolic OR diastolic OR SBP OR DBP)

AND

(randomized OR randomised OR "randomized controlled trial" OR "randomised controlled trial" OR RCT OR trial)

# Funnel plot: IF on BMI in women with overweight or obesity


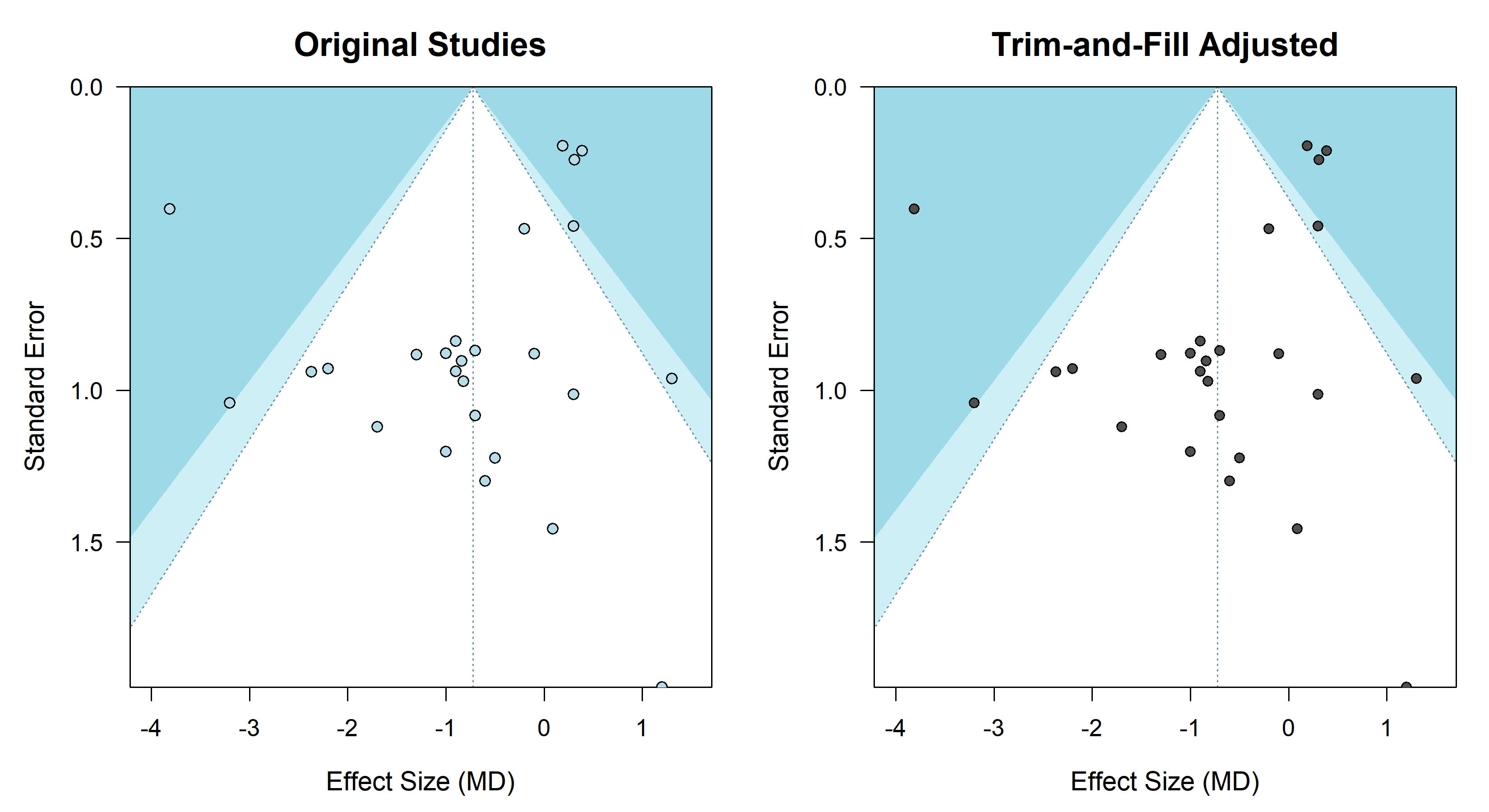


# Sensitivity analysis: IF and BMI


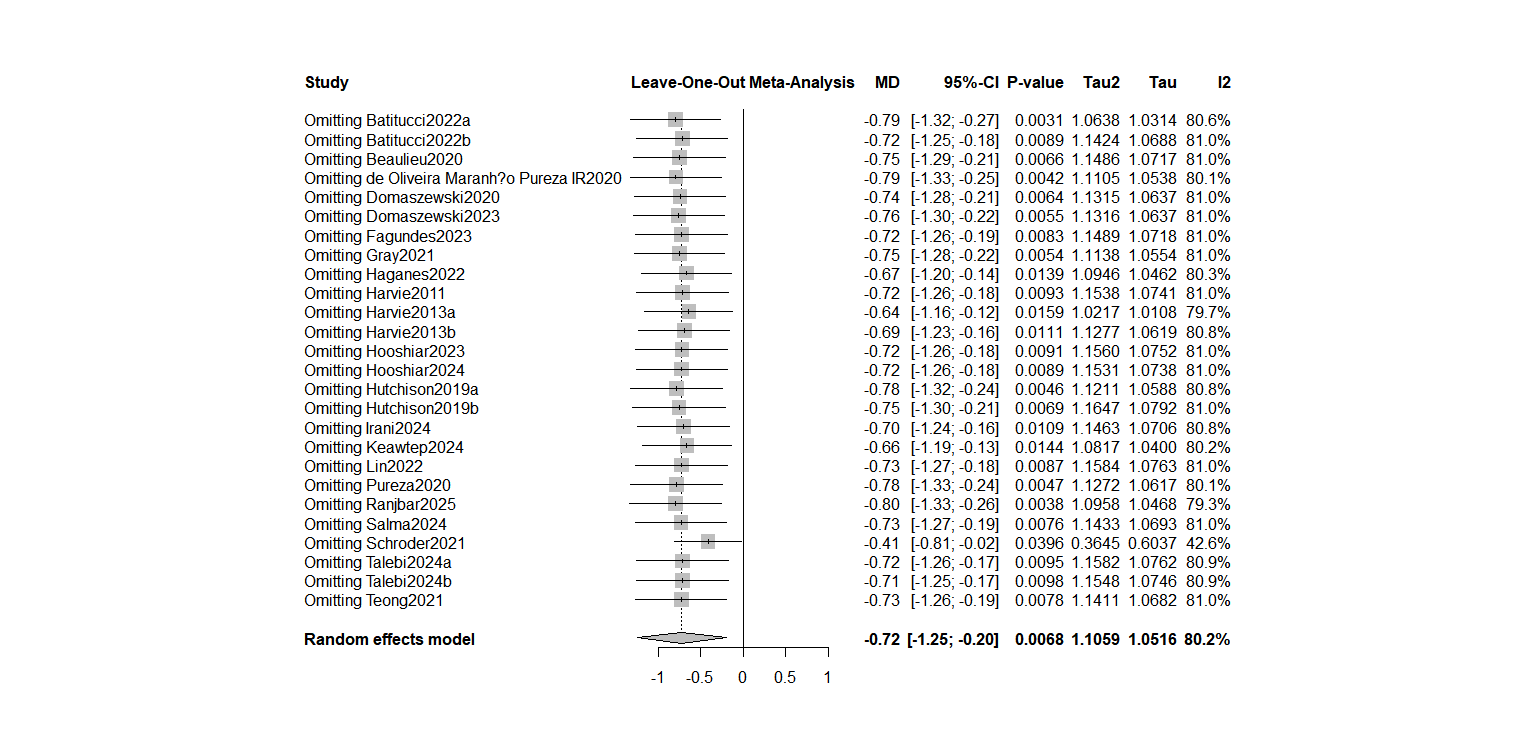


# Funnel plot: IF on fasting blood glucose in women with overweight or obesity


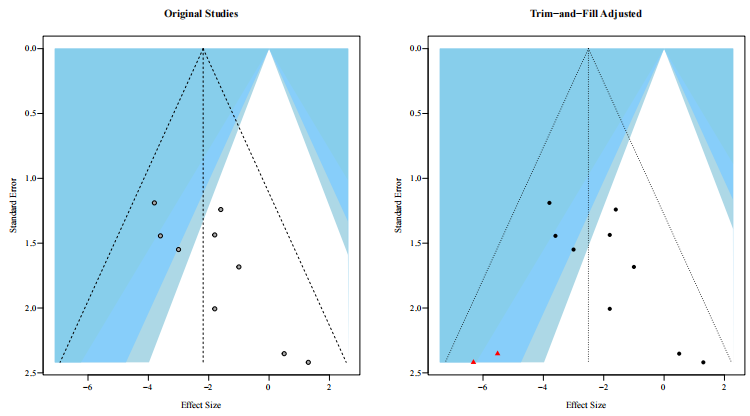


# Sensitivity analysis: IF and fasting blood glucose


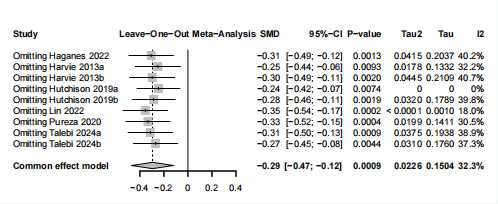


# Funnel plot: IF on systolic blood pressure in women with overweight or obesity


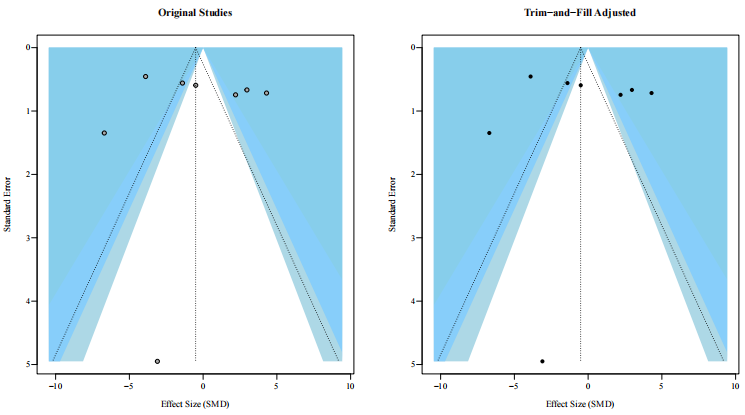


# Sensitivity analysis: IF and systolic blood pressure


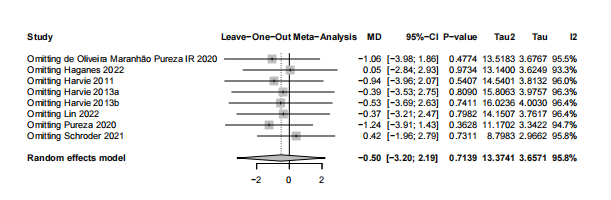


Funnel plot: IF on diastolic blood pressure in women with overweight or obesity


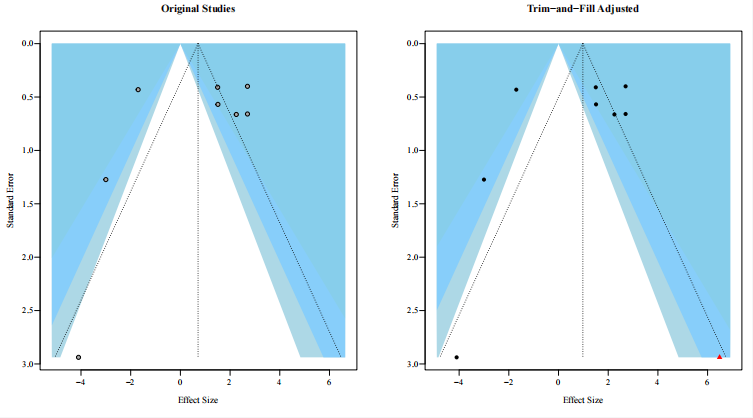


# Sensitivity analysis: **IF** and diastolic blood pressure


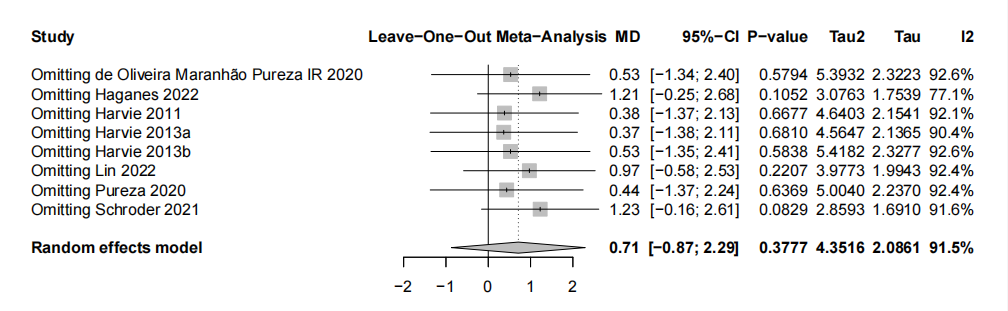


# Supplementary Table S1. Reasons for records removed for other documented reasons before title/abstract screening (n = 157)

| **Category** | **Description** | **n** |
| --- | --- | --- |
| Non-English records | Records published in languages other than English | 37 |
| Ineligible publication type | Conference abstracts, editorials, commentaries, letters, opinion papers, protocols, reviews, and other publication types not eligible for inclusion | 45 |
| Preclinical or non-human studies | Animal studies, in vitro studies, and other preclinical records | 34 |
| Other documented reasons | Other reasons documented during pre-screening data cleaning | 41 |
| Total | 157 | |

# Supplementary Table S2. Full-text reports excluded with reasons (n = 3969)

| **Reason for exclusion at full-text review** | **Explanation** | **n** |
| --- | --- | --- |
| Did not meet inclusion criteria | The full-text report did not satisfy the predefined eligibility criteria after detailed assessment, such as ineligible population, intervention, study design, or outcome scope based on the prespecified PICOS framework. | 2766 |
| Incomplete data or unclear reports | Outcome data were unavailable, incompletely reported, unclearly presented, or could not be extracted for quantitative synthesis. | 981 |
| No eligible control group | The study did not include an eligible control/comparator condition for the planned synthesis. | 222 |
| **Total** | 3969 | |
